# Dysregulation of Cellular VRK1, BAF, and Innate Immune Signaling by the Vaccinia Virus B12 Pseudokinase

**DOI:** 10.1128/jvi.00398-22

**Published:** 2022-05-11

**Authors:** Alexandria C. Linville, Amber B. Rico, Helena Teague, Lucy E. Binsted, Geoffrey L. Smith, Jonas D. Albarnaz, Matthew S. Wiebe

**Affiliations:** a Nebraska Center for Virology, University of Nebraska, Lincoln, Nebraska, USA; b School of Veterinary Medicine and Biomedical Sciences, University of Nebraska, Lincoln, Nebraska, USA; c School of Biological Sciences, University of Nebraska, Lincoln, Nebraska, USA; d Department of Pathology, University of Cambridge, Cambridge, United Kingdom; University of Illinois at Urbana-Champaign

**Keywords:** BAF, innate immunity, poxvirus, protein kinases, vaccinia

## Abstract

Poxvirus proteins remodel signaling throughout the cell by targeting host enzymes for inhibition and redirection. Recently, it was discovered that early in infection the vaccinia virus (VACV) B12 pseudokinase copurifies with the cellular kinase VRK1, a proviral factor, in the nucleus. Although the formation of this complex correlates with inhibition of cytoplasmic VACV DNA replication and likely has other downstream signaling consequences, the molecular mechanisms involved are poorly understood. Here, we further characterize how B12 and VRK1 regulate one another during poxvirus infection. First, we demonstrate that B12 is stabilized in the presence of VRK1 and that VRK1 and B12 coinfluence their respective solubility and subcellular localization. In this regard, we find that B12 promotes VRK1 colocalization with cellular DNA during mitosis and that B12 and VRK1 may be tethered cooperatively to chromatin. Next, we observe that the C-terminal tail of VRK1 is unnecessary for B12-VRK1 complex formation or its proviral activity. Interestingly, we identify a point mutation of B12 capable of abrogating interaction with VRK1 and which renders B12 nonrepressive during infection. Lastly, we investigated the influence of B12 on the host factor BAF and antiviral signaling pathways and find that B12 triggers redistribution of BAF from the cytoplasm to the nucleus. In addition, B12 increases DNA-induced innate immune signaling, revealing a new functional consequence of the B12 pseudokinase. Together, this study characterizes the multifaceted roles B12 plays during poxvirus infection that impact VRK1, BAF, and innate immune signaling.

**IMPORTANCE** Protein pseudokinases comprise a considerable fraction of the human kinome, as well as other forms of life. Recent studies have demonstrated that their lack of key catalytic residues compared to their kinase counterparts does not negate their ability to intersect with molecular signal transduction. While the multifaceted roles pseudokinases can play are known, their contribution to virus infection remains understudied. Here, we further characterize the mechanism of how the VACV B12 pseudokinase and human VRK1 kinase regulate one another in the nucleus during poxvirus infection and inhibit VACV DNA replication. We find that B12 disrupts regulation of VRK1 and its downstream target BAF, while also enhancing DNA-dependent innate immune signaling. Combined with previous data, these studies contribute to the growing field of nuclear pathways targeted by poxviruses and provide evidence of unexplored roles of B12 in the activation of antiviral immunity.

## INTRODUCTION

Protein pseudokinases are becoming increasingly appreciated for their critical roles in governing molecular signal transduction. Classified by their lack of key catalytic residues compared to their kinase counterparts, pseudokinases comprise ~10% of the human kinome (1) and are present at similar or higher levels in the proteomes of all forms of life (2). Studies of pseudokinases thus far have revealed the diversity of functions they may play, including acting as allosteric regulators of enzymes, serving as scaffolds for the assembly of signaling complexes, or regulating the cellular localization or trafficking of proteins (2–4). While pseudokinases play crucial regulatory functions in many biological contexts, their mechanisms of action during infection remain understudied. The study of the product of the vaccinia virus (VACV) *B12R* gene, the B12 pseudokinase, thus provides a valuable opportunity to contribute to the continued elucidation of pseudokinase function.

VACV is a member of the *Orthopoxvirus* genus of the *Poxviridae*, a family of large, enveloped viruses that replicate their ~200-kb double-stranded DNA (dsDNA) genomes entirely in the cytoplasm (5). Homologs of the early VACV *B12* gene are conserved within the orthopoxvirus genus, albeit truncated in variola virus, and no homolog of B12 is found outside the orthopoxvirus genus (6). Broader evolutionary comparisons indicate that B12 is also closely related to the VACV B1 kinase and to a family of three cellular kinases referred to as the Vaccinia-Related Kinases (VRKs) (7). Early studies of B12 characterized the protein as catalytically inactive and apparently nonessential for VACV replication *in vitro* and in murine models of infection (8–10). However, our laboratory discovered an unexpected capability of B12 to act as a repressor of VACV DNA replication in the absence of the VACV B1 kinase (6). These data revealed the existence of a B1-B12 kinase-pseudokinase signaling axis (11), suggesting that phenotypic consequences of B12 function may have been masked during infection in previous models studied that included B1. In addition, B12-mediated inhibition of VACV DNA replication has been determined to be cell type specific and influenced by the levels of host VRK2 protein, arguing that both viral and cellular enzymes modulate how B12 impacts on infection (11). More recently, investigation into the mechanism of action for B12 revealed the formation of a complex containing B12 and the host VRK1 kinase (11).

VRK1 is a member of the mammalian VRK family (7) comprised of two Ser/Thr kinases, VRK1, and VRK2, and the pseudokinase VRK3. VRK1 is an abundant nuclear kinase found in all human cells (12–14) that has been linked to phosphorylation and regulation of transcription factors (15–17), histones (18, 19), and the response to DNA damage (12, 20, 21). In addition, one of VRK1’s most well-defined roles is the direct phosphorylation of BAF to facilitate nuclear envelope disassembly during mitosis, a pathway that is conserved in multiple organisms (22–24). In regards to broad biological importance, VRK1 gene overexpression is found in various types of cancer and is associated with poor prognosis in breast cancer (25–28). In addition, mutations within VRK1 have been characterized in several types of motor neuron diseases (29–33). The mechanism detailing VRK1 contribution to these disease processes remains elusive, and much remains unknown regarding how VRK1 itself is modulated. However, a growing body of evidence indicates that an ~60-amino-acid region C-terminal of its catalytic domain governs nuclear localization and may serve as a docking site for upstream regulators of this kinase (34, 35).

During VACV infection, both VRK1 and VACV B1 kinase phosphorylate and inhibit the antiviral activity of the DNA binding protein BAF (11, 36). Unphosphorylated BAF binds and cross-bridges DNA and thereby inhibits VACV DNA replication, but phosphorylation overcomes this host antiviral defense (11, 36–38). Recent studies have demonstrated that, due to its high affinity for DNA, cytoplasmic BAF limits innate immune signaling leading to expression of type I interferon (IFN) downstream of viral and endogenous DNA sensing (39, 40). However, cells sense virus-derived molecules (pathogen-associated molecular patterns [PAMPs]) through a variety of pattern recognition receptors (PRRs) that activate the production of type I IFNs and other proinflammatory molecules (41). Type I IFNs stimulate the expression of hundreds of IFN-stimulated genes (ISGs), whose products display a wide range of virus-restricting activities (42).

In this study, we further characterize functional linkages between B12 and cellular pathways involving VRK1, BAF, and innate immune signaling. We include mutational analysis of both B12 and VRK1, providing a better understanding of how B12 inhibits the proviral activity of VRK1. We also find clear evidence of mutual regulation of B12 and VRK1, including stabilization of B12 by VRK1 and the subcellular relocalization of both proteins. Next, we demonstrate that B12 also impacts the localization of BAF, causing BAF to repartition predominantly to the nucleus in a manner correlating with reduced BAF phosphorylation. Lastly, we discover that B12 increases innate immune signaling induced by DNA stimulation, indicating a new functional consequence of the B12 pseudokinase. This study expands upon the previous identification of a functional complex formed between B12 and VRK1 with new evidence demonstrating the diverse signaling impacts of B12.

## RESULTS

### VRK1 influences the stability of B12.

**FIG 1:**
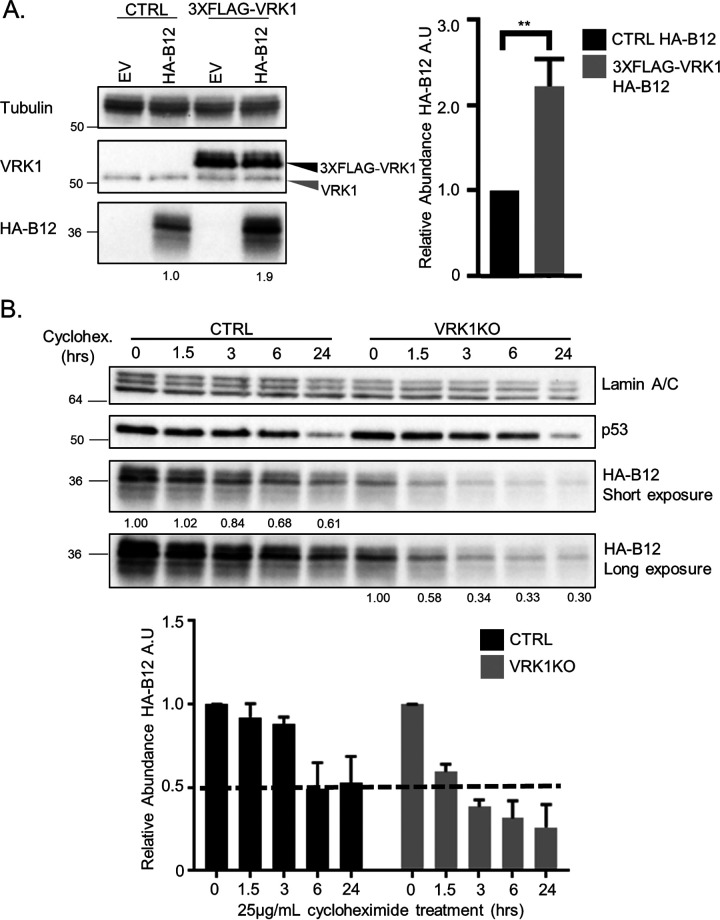
VRK1 influences the stability of B12. (A) Immunoblot analyses of whole-cell lysates from CV1 cells transduced with empty vector (CTRL)-, 3×FLAG-VRK1-, and HA-B12-expressing lentiviruses. Quantitation of HA-B12 abundance in CV1 transduced cells was performed over three independent replicates and two independent cell generations and normalized to tubulin detection. A representative immunoblot shown (**, *P* < 0.01). (B) Immunoblot analyses of whole-cell lysates from HAP1 or HAP1-VRK1KO cells transduced with HA-B12-expressing lentivirus and treated with 25 μg/mL cycloheximide over a time course. Quantitation of HA-B12 abundance and half-life was performed over three independent replicates and normalized to lamin A/C protein levels. A representative immunoblot is shown.

Our previous study demonstrated that B12 coprecipitates VRK1 in VACV-infected or uninfected cells and influences events downstream of VRK1 (11). We sought to further investigate the properties of the B12/VRK1 complex, hypothesizing that B12 activity during VACV infection was linked to complex formation and regulation of VRK1. Examining hemagglutinin-tagged B12 (HA-B12) steady-state protein levels in CV1 cells overexpressing 3×FLAG-VRK1 revealed that HA-B12 levels were increased significantly (2-fold, Fig. 1A) in the presence of increased amounts of VRK1 compared to control CV1s, while VRK1 levels were unaffected by the presence of HA-B12. To further investigate VRK1’s influence on the stability of B12, we examined the half-life of HA-B12 in normal and VRK1KO (knockout) HAP1 cells during cycloheximide-induced translation inhibition. In the presence of VRK1 (CTRL cells), the half-life of HA-B12 was greater than 6 h (hours) (Fig. 1B), but in the absence of VRK1 (VRK1KO cells), it was less than 3 h (Fig. 1B). We also observed markedly less total HA-B12 protein at the initial time point (0 h) in VRK1KO cells compared to CTRL HAP1 cells (Fig. 1B). These trends were specific to HA-B12 because the apparent half-lives of control proteins lamin A/C and p53 were unaffected by the presence or absence of VRK1. These results indicate that VRK1 increases the stability and steady-state levels of B12, potentially through their complex formation.

### VRK1 and B12 coinfluence their solubility properties.

**FIG 2:**
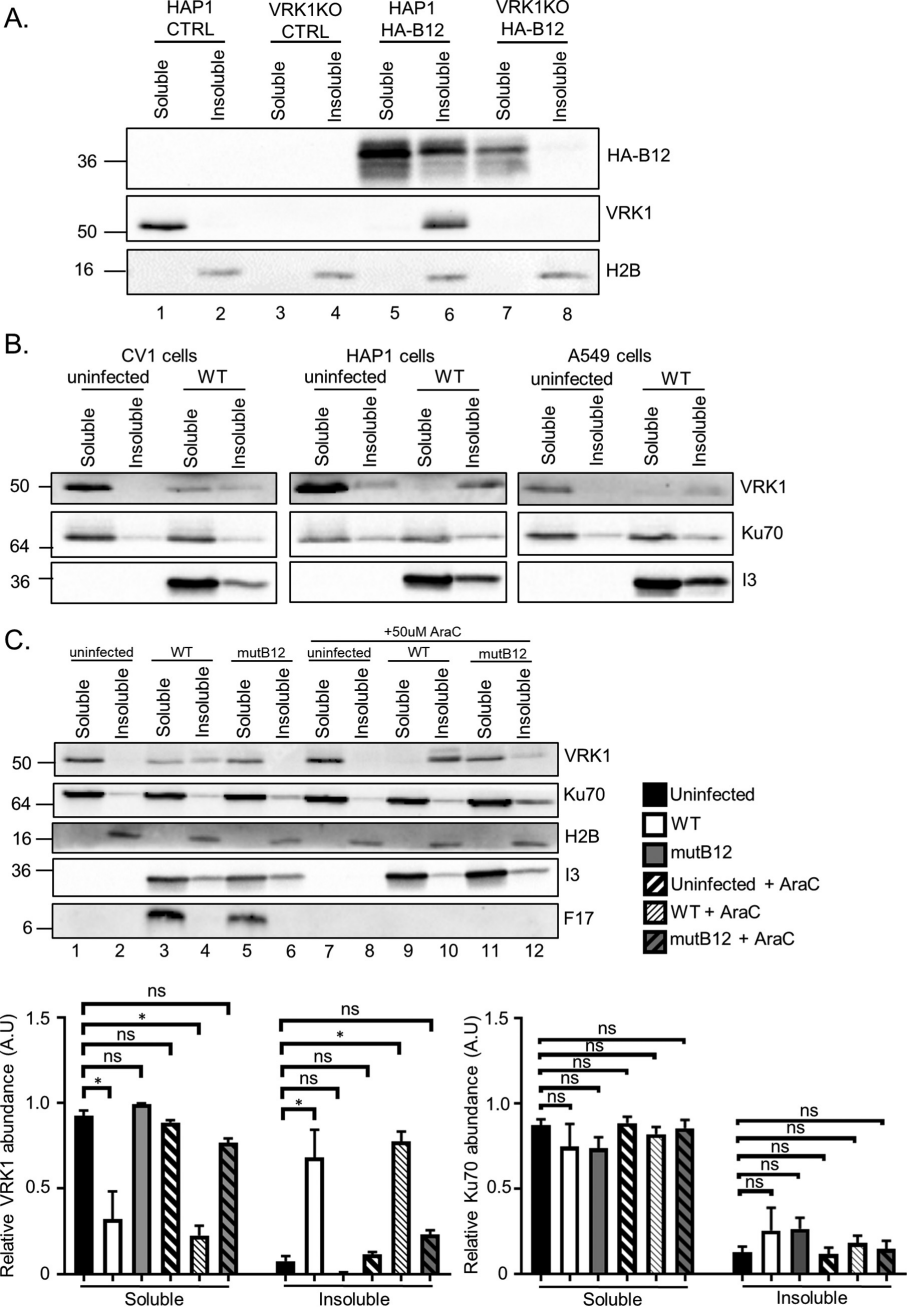
VRK1 and B12 coinfluence their solubility properties. (A) Immunoblot analyses of detergent-treated fractions from HAP1 or HAP1-VRK1KO cells transduced with empty-vector (CTRL)- or HA-B12-expressing lentiviruses. Immunoblots detecting Ku70 and histone 2B are shown as fractionation controls. (B) Immunoblot analyses of detergent fractions from CV1, HAP1, or A549 cells uninfected or infected with WT VACV (WR) at an MOI of 5 and harvested at 7 hpi. (C) Immunoblot analyses of fractions from A549 cells uninfected or infected with WT or mutB12 virus (MOI = 5), treated with or without 50 μM AraC, and harvested at 7 hpi. Quantitation of VRK1 and Ku70 abundances was performed for each fraction, with each combined sample abundance (soluble + insoluble) set to 1. Three independent replicates were quantified, (*, *P* < 0.05). A representative immunoblot is shown.

To further investigate the cellular properties of both proteins, we examined the solubility of B12 in the presence or absence of VRK1. In this assay, the soluble fraction represents proteins released with non-ionic detergent treatment, while the insoluble fraction represents proteins sedimented by centrifugation. Ku70 and histone 2B were selected as examples of a predominantly nucleoplasmic and chromatin-bound protein, respectively, for these assays. We found that in the absence of VRK1, HA-B12 decreases in abundance in the soluble fraction (Fig. 2A, compare lanes 5 and 7), and is absent from the insoluble fraction (Fig. 2A, compare lanes 6 and 8) compared to control cells. Further, in the absence of HA-B12, VRK1 was found only in the soluble fraction (Fig. 2A), but in the presence of HA-B12, VRK1 was shifted completely to the insoluble fraction (Fig. 2A). These results indicate that B12 and VRK1 influence the solubility profiles of one another, suggesting that a B12/VRK1 complex may be less soluble under these conditions than either protein alone.

To determine whether the above changes in VRK1 fractionation also occur during VACV infection and in other cell types, we examined VRK1 solubility in uninfected versus VACV-infected CV1, HAP1, and A549 cells. By 7 h postinfection (hpi), wild-type (WT) virus infection shifted VRK1 from highly soluble in uninfected cells to a more insoluble state (Fig. 2B). This was seen in all cell lines, although less pronounced in CV1 cells, and was not due to a general shift in protein solubility because Ku70 solubility (Fig. 2B) remained unchanged. To test whether this solubility shift was mediated by B12 and to examine the influence of viral DNA replication and late viral proteins, the experiment was repeated with mutB12 (virus lacking a functional *B12R* gene) and with or without the viral DNA replication inhibitor AraC. Strikingly, the decrease in VRK1 solubility during WT infection was lost with mutB12 infection (Fig. 2C, compare lanes 4 and 6). Immunoblots of the early viral protein I3 and late viral protein F17 indicate similar infection by the WT and mutB12 viruses. A quantitative analysis of VRK1 fractionation across multiple experiments is shown at the bottom of Fig. 2C. Only 30 to 50% of VRK1 remained soluble during WT infection, whereas in uninfected or mutB12-infected cells, 95 to 100% of VRK1 was soluble (Fig. 2C, bottom left). Again, these solubility changes are specific to VRK1, because the distribution of Ku70 was unchanged (Fig. 2C, bottom right). Lastly, viral DNA replication and late viral protein expression did not impact the solubility of VRK1 (Fig. 2C). In conclusion, these data demonstrate that B12 is necessary and sufficient to repartition VRK1.

### B12 and VRK1 coinfluence their chromatin subcellular localization.

**FIG 3:**
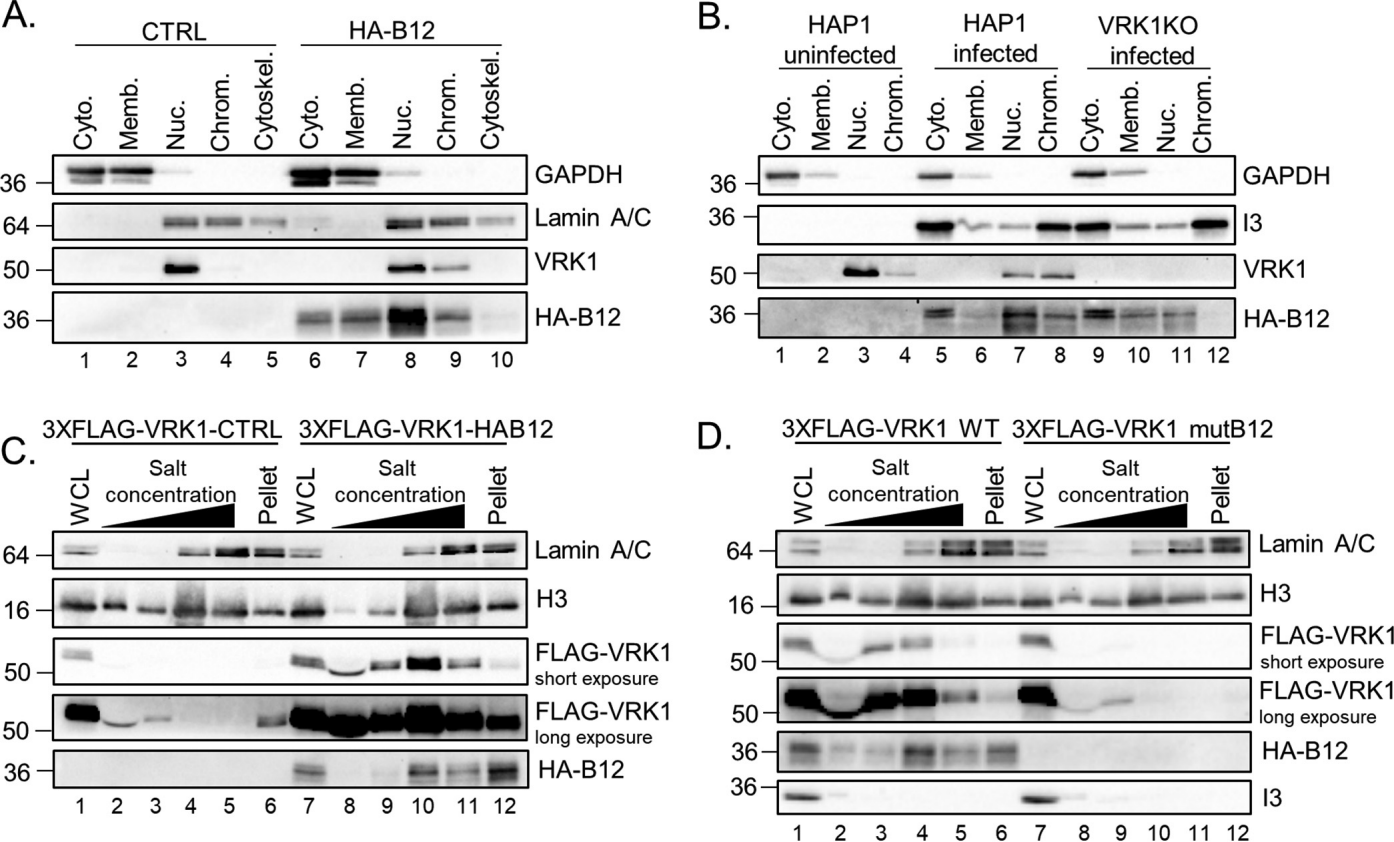
B12 and VRK1 coinfluence their chromatin subcellular localization. (A) Immunoblot analyses of subcellular fractions from CV1 cells transduced with empty-vector (CTRL)- or HA-B12-expressing lentiviruses. (B) Immunoblot analyses of subcellular fractions from HAP1 or HAP1-VRK1KO cells uninfected or infected with WTeHAB12 virus (MOI = 5) and harvested at 7 hpi. (C) Immunoblot analyses of nuclei salt fractions from HAP1-VRK1KO cells transduced with lentiviruses expressing empty-vector (CTRL), 3×FLAG-VRK1, or HA-B12. (D) Immunoblot analyses of nuclear salt fractions from HAP1-VRK1KO cells transduced with empty-vector (CTRL)- or 3×FLAG-VRK1-expressing lentiviruses, infected with WT or mutB12 virus (MOI = 5), treated with 50 μM AraC, and harvested at 7 hpi.

To investigate whether the B12-mediated change in VRK1 solubility affected protein localization, the distribution of VRK1 was studied by subcellular fractionation. In these assays, VRK1 was found exclusively in the soluble nuclear fraction (Fig. 3A, lane 3) in control (CTRL) CV1 cells, but in the presence of B12 it was also detected in the chromatin-associated nuclear fraction (Fig. 3A, lanes 8 and 9). This VRK1 fractionation profile overlaps considerably with that of HA-B12 (Fig. 3A, lanes 6 to 9) as reported earlier (6). To determine whether these changes also occurred during VACV infection, and to investigate how the absence of VRK1 influences the localization of HA-B12, this subcellular fractionation assay was performed using uninfected HAP1 cells, as well as HAP1 and HAP1-VRK1KO cells infected with WTeHAB12 virus. The WTeHAB12 virus expresses B12 with an N-terminal HA-tag from its endogenous locus to enable B12 detection during infection. Similar to the CV1 cells, in uninfected HAP1 cells, VRK1 was found predominantly in the soluble nuclear fraction (Fig. 3B, lanes 3 and 4), while WT infection results in less VRK1 detection in the soluble nuclear fractionation and greater detection in the chromatin-associated nuclear fraction (Fig. 3B, lanes 7 and 8). Interestingly, in the absence of VRK1, HA-B12 localization was decreased in the soluble nuclear fraction (Fig. 3B, compare lanes 7 and 11), and completely absent from the chromatin-associated nuclear fraction (Fig. 3B, compare lanes 8 and 12). Taken together, these data demonstrate that the chromatin-associated localization of VRK1 and B12 are codependent on their shared presence.

To further investigate the chromatin-associated localization of VRK1 and B12, we examined salt fractionation profiles of isolated nuclei using established protocols (43–45). This assay allows for a dissection of the composition of nuclear proteins complexes as assessed by their fractionation in the presence of increasing salt concentrations. In the absence of HA-B12, 3×FLAG-VRK1 is almost undetectable in nuclei isolated following cell lysis with nonionic detergent (Fig. 3C, lane 2), consistent with the absence of VRK1 from the insoluble fraction of nonionic detergent-treated cells in Fig. 2. In stark contrast, HA-B12 relocalizes 3×FLAG-VRK1 in the nucleus, increasing its abundance in low salt fractions (Fig. 3C, compare lanes 2 and 3 to lanes 8 and 9) and resulting in robust detection in higher salt fractions, with the highest abundance in the same salt concentration as that enriched in histone 3 (H3) (Fig. 3C, lane 10). Similar B12-dependent trends were seen with nuclei of WTeHAB12- or mutB12-infected cells that underwent salt fractionation. Specifically, in contrast with WT VACV-infected cells, 3×FLAG-VRK1 was absent in higher salt nuclear fractions during infection with mutB12, lacking functional B12 (Fig. 3D, compare lanes 4 and 10). In conclusion, these results indicate that B12 influences VRK1 intranuclear detection, redirecting VRK1 to a chromatin-associated complex.

### B12 induces VRK1 retention on chromatin during mitosis.

**FIG 4:**
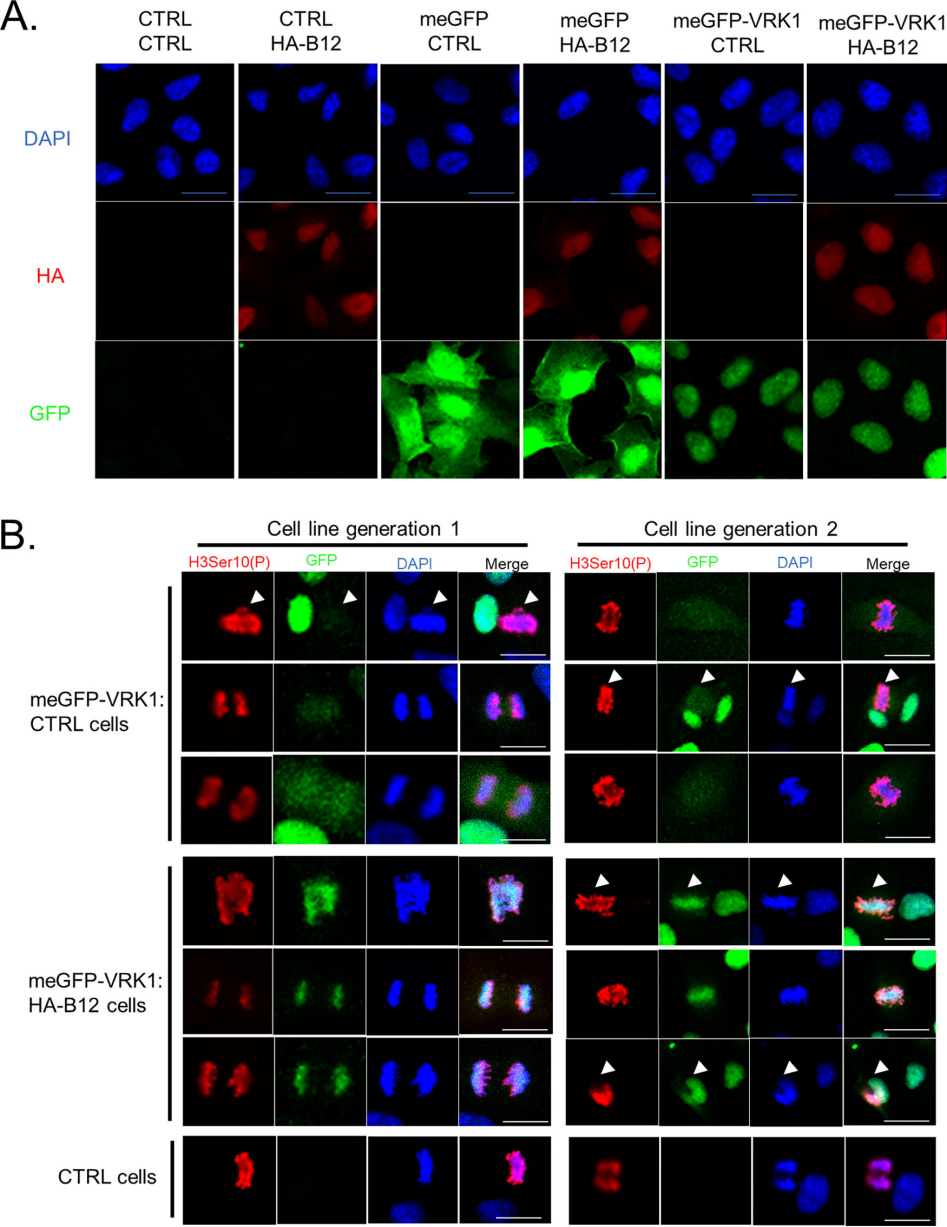
B12 maintains VRK1 on chromatin during mitosis. (A) Immunofluorescence of CV1 cells transduced with empty-vector (CTRL)-, meGFP-, meGFP-VRK1-, and HA-B12-expressing lentiviruses. DAPI (nuclei), HA (B12), and GFP (alone or tagged to VRK1) were detected. (B) Immunofluorescence of mitotic CV1 cells transduced with empty vector (CTRL)-, meGFP-VRK1-, and HA-B12-expressing lentiviruses. Representative images from independent cell generations are shown. DAPI (DNA), H3Ser10(P), and GFP (VRK1) were detected. Arrow bars present in some panels to distinguish mitotic cells. Scale bar, 20 μm.

To corroborate the observation that B12 enhances chromatin association of VRK1, we examined the localization of VRK1 by immunofluorescence microscopy. We generated an N-terminal meGFP (monomeric enhanced green fluorescent protein)-tagged VRK1 and stable expression in CV1 cells resulted in nuclear detection of meGFP-VRK1, in agreement with studies conducted with a dsRED-tagged VRK1 (18), while meGFP alone was more diffuse within the cell (Fig. 4A). Nuclear detection of meGFP-VRK1 was also observed with stable expression in HA-B12-expressing CV1 cells (Fig. 4A), with no clear differences in VRK1’s intranuclear localization seen in interphase cells. The predominantly nuclear localization of HA-B12 was also unchanged with meGFP or meGFP-VRK1 expression (Fig. 4A). While we did not observe a difference in meGFP-VRK1 localization with or without HA-B12 in interphase cells, we hypothesized that we might capture relocalization of VRK1 by B12 to chromatin in mitotic cells. Mitotic cells provide an opportunity to visualize compacted chromatin while also releasing the majority of nuclear proteins not associated with the chromatin to mix with the cytoplasm upon dissolution of the nuclear envelope. The release of such proteins, including VRK1 (13), results in diffuse detection of these proteins during mitosis. To identify mitotic cells in an asynchronous population, we used a H3Ser10(P)-specific antibody along with DAPI staining of compacted chromatin. In control CV1 cells, meGFP-VRK1 is localized diffusely in mitotic cells with no distinct overlap with DAPI chromatin staining or H3Ser10(P) signal (Fig. 4B, top panel, arrowheads are used to distinguish mitotic cells in some panels). However, in HA-B12-expressing CV1 cells, meGFP-VRK1 localization markedly overlaps with DAPI and H3Ser10(P) staining (Fig. 4B, bottom panel). These localization patterns were observed in independent cell transductions (Fig. 4B, compare left and right panels). Taken together, these results demonstrate that B12 drives the relocalization of VRK1 to chromatin, a phenotype that can be clearly visualized in mitotic cells.

### VRK1 and B12 influence each other’s subcellular localization.

**FIG 5:**
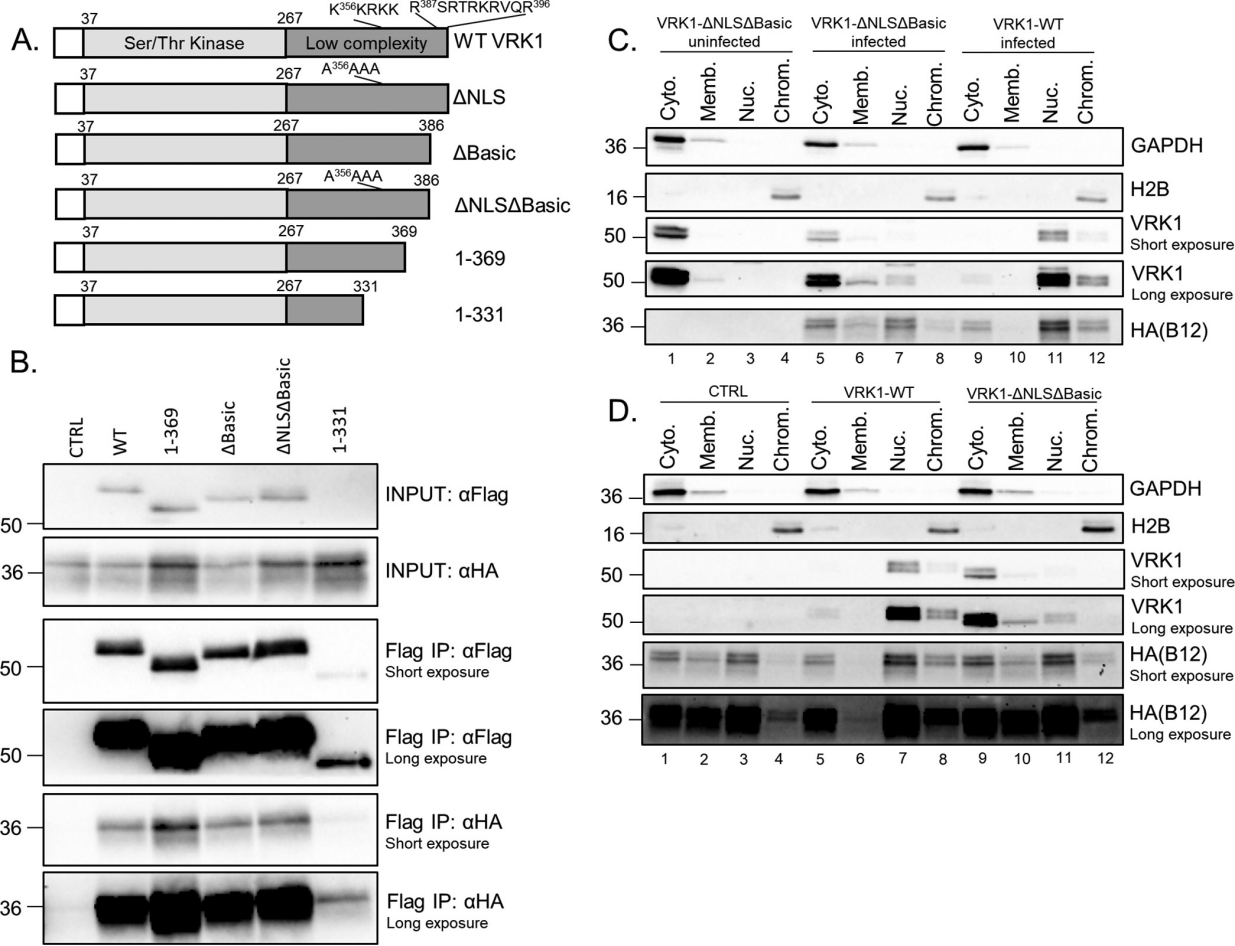
VRK1 and B12 dynamically influence each other’s subcellular localization. (A) Depiction of panel of N-terminal 3×FLAG-human VRK1 mutant constructs. (B) Immunoblot analyses of INPUT and immunoprecipitated lysates from HAP1-VRK1KO cells transduced with empty-vector (CTRL)-, 3×FLAG-VRK1 (WT)-, 3×FLAG-VRK1 (1-369)-, 3×FLAG-VRK1 (ΔBasic)-, 3×FLAG-VRK1 (ΔNLSΔBasic)-, or 3×FLAG-VRK1 (1-331)-expressing lentiviruses and infected with WR-HAB12 virus (MOI = 5), harvested at 7 hpi, and subjected to FLAG-specific immunoprecipitation. (C) Immunoblot analyses of subcellular fractions from HAP1-VRK1KO cells transduced with 3×FLAG-VRK1 (ΔNLSΔBasic)- or 3×FLAG-VRK1 (WT)-expressing lentiviruses and uninfected or infected with WTeHAB12 (MOI = 5) and harvested at 7 hpi. (D) Immunoblot analyses of subcellular fractions from HAP1-VRK1KO cells transduced with empty-vector (CTRL)-, 3×FLAG-VRK1 (WT)-, or 3×FLAG-VRK1 (ΔNLSΔBasic)-expressing lentiviruses, infected with WTeHAB12 (MOI = 5), and harvested at 7 hpi.

Given the influence of B12 and VRK1 on their chromatin-associated subcellular localization, we investigated if the nuclear localization of VRK1 was necessary to form a complex with B12 and influence its subcellular localization. The structure of VRK1 has been solved and is composed of two main domains, an N-terminal Ser/Thr kinase catalytic domain and a C-terminal low-complexity, noncatalytic tail (46). Within the C-terminal tail are two proposed nuclear localization sites (NLS) (7), a stretch of lysine and arginine residues beginning at amino acid 356 and a stretch of charged “basic” residues beginning at amino acid 387. In addition, the site of interaction between some VRK1-interacting proteins has been mapped to its C-terminal tail, including H3, p53, and RanGTP (17, 18, 34). A panel of 3×FLAG-tagged VRK1 mutants with mutated NLS sites and C-terminal truncations was constructed (Fig. 5A), and their interactions with B12 were examined by immunoprecipitation. These mutants were expressed at similar levels to WT VRK1, except for the 1-331 mutant that was less abundant (Fig. 5B, INPUT αFlag). The low abundance of the 1-331 mutant was expected, given the reported importance of the C-terminal tail for VRK1 stability (46). The 3×FLAG-VRK1 mutants 1-369, ΔBasic, and ΔNLSΔBasic coimmunoprecipitated B12 to levels equivalent to WT VRK1 (Fig. 5B, Flag IP: αHA short exposure). Even the low-abundance 3×FLAGVRK1 1-331 mutant coimmunoprecipitated B12 measurably (Fig. 5B, Flag IP: αHA long exposure). Thus, disrupting the NLS sites and C-terminal tail of VRK1 did not abrogate its ability to form a complex with B12, suggesting the region(s) responsible for the coprecipitation lie outside these regions.

Given that B12 retains the ability to coprecipitate with the VRK1-ΔNLSΔBasic mutant, we tested whether the nuclear targeting sequences of VRK1 are necessary for the localization of B12 to the chromatin. Whereas VRK1-ΔNLSΔBasic is predominantly cytoplasmic in uninfected cells, this mutant shifted partially to the membrane-associated and soluble nuclear fractions upon WTeHAB12 infection (Fig. 5C, compare lanes 1 to 3 to lanes 5 to 7). However, unlike WT VRK1, infection was not sufficient to relocalize VRK1-ΔNLSΔBasic to the chromatin fraction under these conditions (Fig. 5C, compare lanes 8 and 12). Interestingly, the increased localization of VRK1-ΔNLSΔBasic in the cytoplasm and membrane-associated fractions correlated with an increase in HA-B12 detection in these fractions (Fig. 5C, compare lanes 5 and 6 to lanes 9 and 10). Similarly, the decrease of VRK1-ΔNLSΔBasic in the soluble nuclear and chromatin-associated fractions compared to WT VRK1 correlated with decreased HA-B12 detection in these fractions (Fig. 5C, compare lanes 7 and 8 to lanes 11 and 12). To further examine the impact of VRK1 on B12 localization, we compared the localization of HA-B12 during infection using cells lacking VRK1 (VRK1KO-CTRL) or cells expressing either nuclear VRK1 (VRK1KO-VRK1 WT) or cytoplasmic VRK1 (VRK1KO-VRK1 ΔNLSΔBasic). The reintroduction of WT VRK1 into the VRK1KO cells increased the abundance of HA-B12 in both the soluble nuclear (Fig. 5D, compare lanes 3 and 7) and chromatin-associated nuclear fractions (Fig. 5D, compare lanes 4 and 8). In comparison, the reconstitution of cytoplasmic VRK1-ΔNLSΔBasic into the VRK1KO cells correlated with increased abundance of HA-B12 in the cytoplasmic fraction (Fig. 5D, compare lanes 1 and 9), as well as modestly increasing HA-B12 abundance in the soluble nuclear and chromatin-associated fractions (Fig. 5D, compare lanes 3 and 4 to lanes 11 and 12). Taken together, these results suggest that the complex formed between VRK1 and B12 dictate the subcellular localization of both proteins in a manner independent of NLS segments within the C-terminal region of VRK1.

### The nuclear localization of VRK1 is not required for its proviral function.

**FIG 6:**
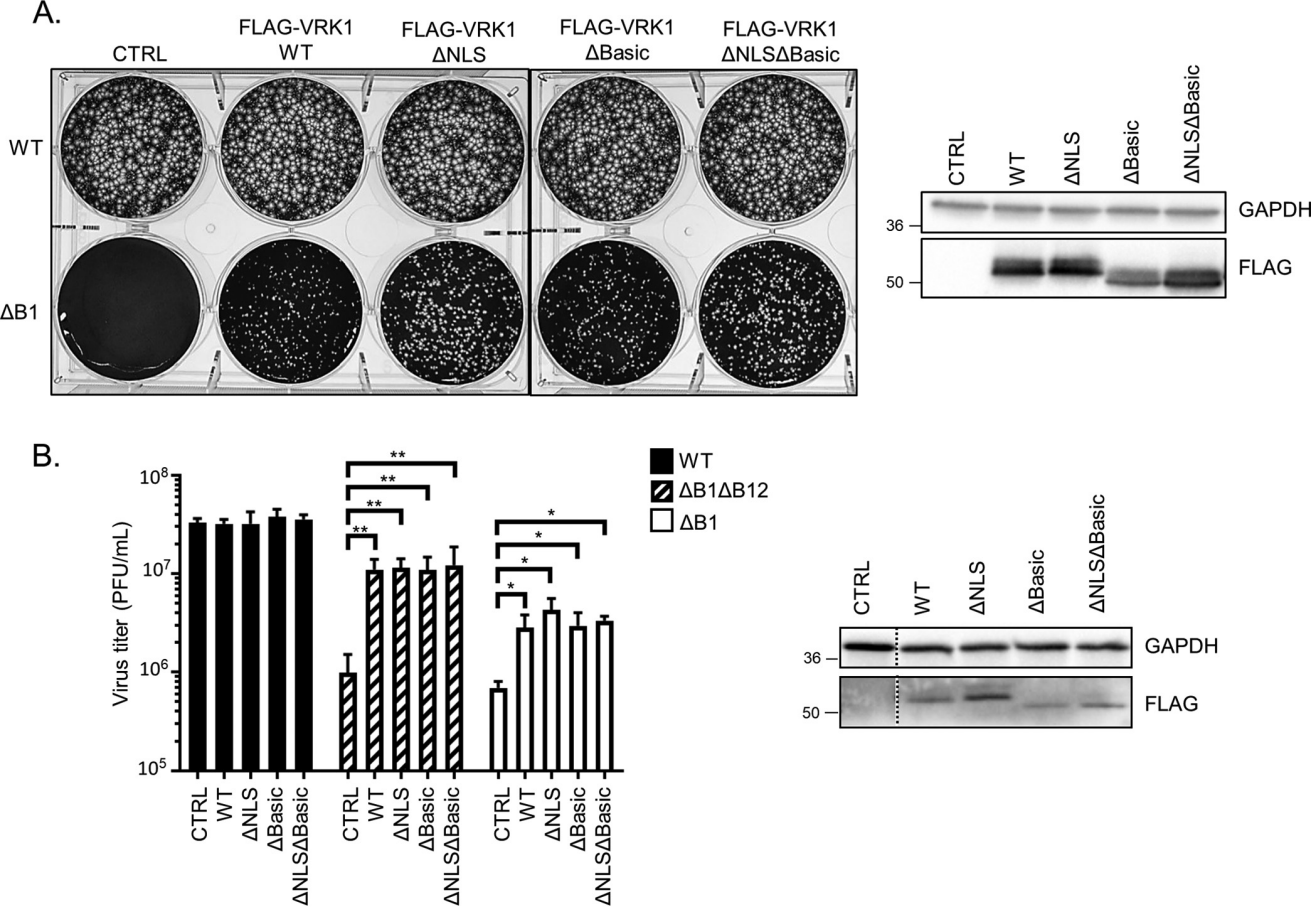
The nuclear localization of VRK1 is not required for its proviral function. (A, left) Plaque assay of CV1 cells transduced with empty-vector (CTRL)-, 3×FLAG-VRK1 (WT)-, 3×FLAG-VRK1 (ΔNLS)-, 3×FLAG-VRK1 (ΔBasic)-, or 3×FLAG-VRK1 (ΔNLSΔBasic)-expressing lentiviruses, infected with 300 PFU of WT or ΔB1 viruses, and fixed at 72 hpi with crystal violet/paraformaldehyde solution. (Right) Immunoblot analyses of whole-cell lysates used for the plaque assay. (B, left) Virus titers from HAP1-VRK1KO cells transduced with empty vector (CTRL)-, 3×FLAG-VRK1 (WT)-, 3×FLAG-VRK1 (ΔNLS)-, 3×FLAG-VRK1 (ΔBasic)-, or 3×FLAG-VRK1 (ΔNLSΔBasic)-expressing lentiviruses and infected with WT, ΔB1ΔB12, or ΔB1 viruses (MOI = 3; *, *P* < 0.05; **, *P* < 0.01). (Right) Immunoblot analyses of whole-cell lysates used for virus infections.

Our previous study demonstrated that exogenous VRK1 expression was sufficient to increase ΔB1 virus fitness and was necessary for rescue of the ΔB1mutB12 virus (11). As the C-terminal tail of VRK1 directs subcellular localization and some protein-protein interactions, we investigated if some of its conserved motifs and its nuclear localization were necessary for its proviral function. As shown earlier, mutation of both the NLS and basic regions completely removes the nuclear localization of VRK1 (Fig. 5C), while the single mutation of either the NLS or basic region results in partial relocalization to the cytoplasm (7). Stable expression of VRK1 mutants lacking either the NLS or basic region in CV1 cells resulted in rescue of ΔB1 plaque formation comparable to WT VRK1 (Fig. 6A), albeit with some modest differences in plaque size. Similar results of ΔB1 virus rescue were also obtained in HAP1-VRK1KO cells, in which stable expression of these VRK1 mutants rescued both ΔB1ΔB12 and ΔB1 virus titers to levels similar to WT VRK1 compared to cells lacking VRK1 (Fig. 6B). In conclusion, the NLS C-terminal motifs and the nuclear localization of VRK1 are not required for its proviral ability to rescue ΔB1 viruses.

### Identification of a B12 point mutation that disrupts complex formation with VRK1.

**FIG 7:**
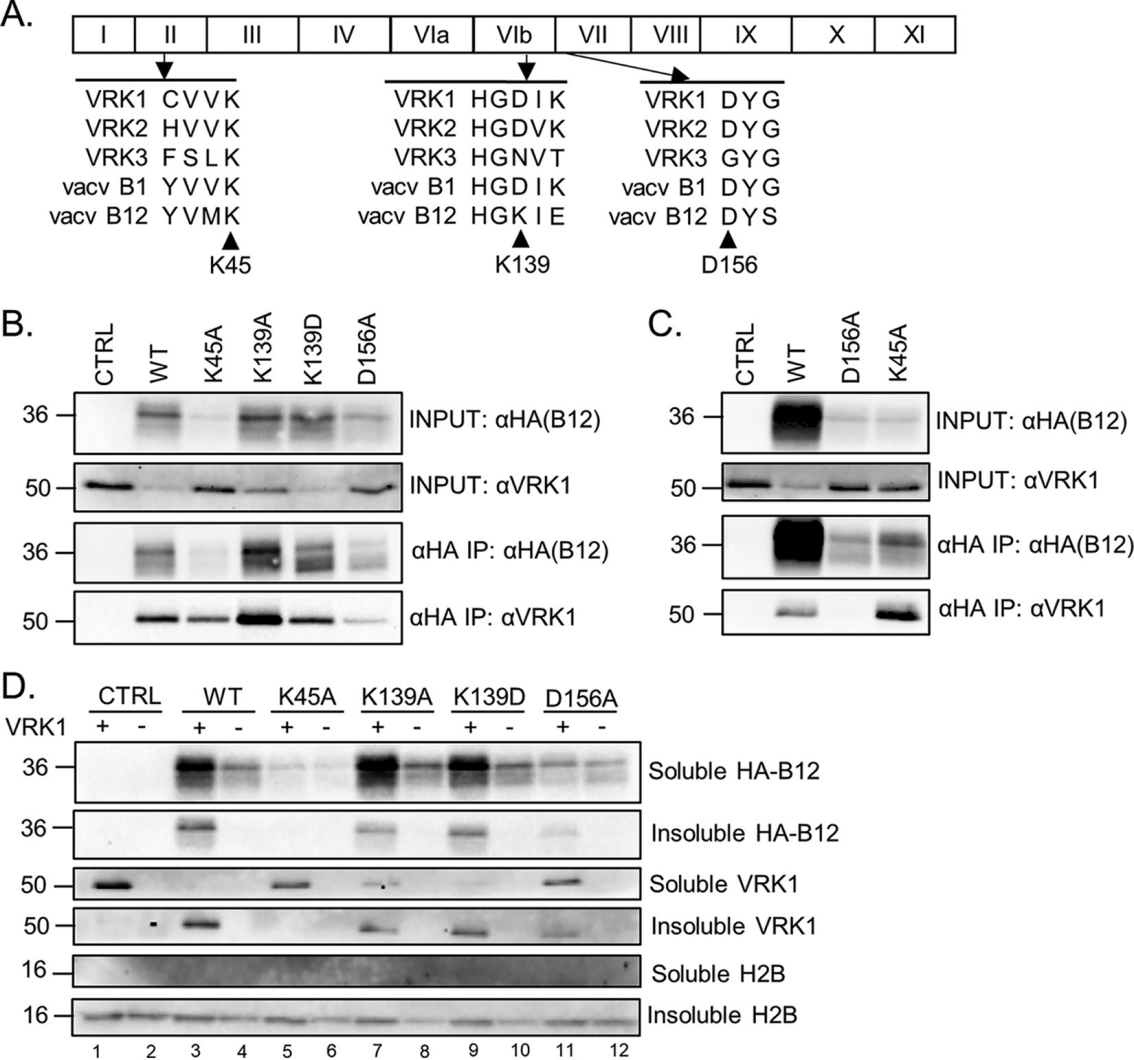
Identification of a B12 point mutation that disrupts complex formation with VRK1. (A) Locations of B12 point mutations based on protein domain homology with other vaccinia virus-related kinase family members. (B) Immunoblot analyses of INPUT and immunoprecipitated lysates from CV1 cells transduced with empty-vector (CTRL)-, HA-B12 (WT)-, HA-B12 (K45A)-, HA-B12 (K139A)-, HA-B12 (K139D)-, or HA-B12 (D156A)-expressing lentiviruses and subjected to HA tag-mediated immunoprecipitation. (C) Immunoblot analyses of INPUT and immunoprecipitated lysates from 293T cells transfected with 2 μg of pcDNA3.1-empty (CTRL), pcDNA3.1-HA-B12 (WT), pcDNA3.1-HA-B12 (D156A), or pcDNA3.1-HA-B12 (K45A) expression plasmids, harvested at 48 h posttransfection, and subjected to HA tag-specific immunoprecipitation. (D) Immunoblot analyses of fractions from HAP1 and HAP1-VRK1KO cells transduced with empty-vector (CTRL)-, HA-B12 (WT)-, HA-B12 (K45A)-, HA-B12 (K139A)-, HA-B12 (K139D)-, or HA-B12 (D156A)-expressing lentiviruses.

Next, we sought to identify B12 mutations that disrupt complex formation with VRK1, while recognizing that such mutant(s) would likely accumulate to lower levels than WT B12, given our observations that VRK1 influences B12 stability (Fig. 1). We targeted residues conserved in other homologous kinases, basing our alignment on the high conservation of these regions for classification of serine-threonine protein kinases in domains II, VIb, and VII (47). Domains II and VII of the protein kinase superfamily are essential for ATP binding and stabilization, while domain VIb contains the essential aspartic acid needed for ATP catalysis. This approach led to the construction of four B12 mutants: K45A, K139A, K139D, and D156A (Fig. 7A).

Stable expression of these HA-tagged mutants in CV1 cells and examination of their abundance showed that the K45A and D156A mutants were present at levels below WT, while K139A and K139D mutants were present at levels comparable to WT B12 (Fig. 7B, INPUT αHA). Following immunoprecipitation, the amount of protein isolated for each B12 mutant corresponded well with their relative abundance in the input samples (Fig. 7B, IP αHA). Next, we compared the amount of copurified VRK1 with each mutant. In contrast to the B12 K139A and K139D mutants, which coimmunoprecipitated similar amounts of VRK1 to WT B12, immunoprecipitation of the D156A mutant indicated it was the most clearly disrupted in its capacity to coimmunoprecipitate VRK1 (Fig. 7B, αHA IP: αVRK1). Notably, despite the decreased overall amount of B12-K45A immunoprecipitated (Fig. 7B, αHA IP: αHA), the amount of VRK1 coimmunoprecipitated was at least comparable to WT B12. To corroborate the results of the B12-K45A and B12-D156A mutants in another expression system and cell type, 293T cells were transiently transfected to express HA-tagged B12-WT, B12-K45A, or B12-D156A. While both B12-D156A and B12-K45A were expressed considerably less than WT B12 (Fig. 7C, INPUT αHA) and immunoprecipitated at lower levels (Fig. 7C, αHA IP: αHA), only the B12-D156A mutant demonstrated a clear decrease in coimmunoprecipitation of VRK1 (Fig. 7C, αHA IP: αVRK1).

To investigate whether the ability of these B12 mutants to immunoprecipitate VRK1 correlated with their fractionation profile, we utilized the fractionation protocol used in Fig. 2. We hypothesized that a B12 mutant with disrupted capacity to form a complex with VRK1 would also be unable to shift VRK1 to the insoluble fraction and its fractionation profile would be unaffected in the absence of VRK1. Indeed, upon expression of either the B12-K139A or B12-K139D mutants, we observed a similar shift in VRK1 to the insoluble fraction as occurred during WT B12 expression (Fig. 7D). In addition, the absence of VRK1 similarly decreased B12-K139A and B12-K139D abundance in the soluble fraction and correlated with their loss from the insoluble fraction (Fig. 7D). In contrast, expression of the B12-D156A mutant did not shift VRK1 to the insoluble fraction and its soluble fractionation was unchanged in the absence of VRK1 (Fig. 7D). The data involving B12-K45A is more complex to interpret. Like B12-D156A, B12-K45A was unable to shift VRK1 to the insoluble fraction in this assay compared to WT-B12, even though it can form a complex with VRK1 detectable by coimmunoprecipitation (Fig. 7B and C). It is unclear whether these data indicate that the low levels of B12-K45A are insufficient to relocalize VRK1 or whether it has lost another property needed for the fractionation shift. Taken together, the immunoprecipitation and fractionation results suggest that the D156A mutation of B12 disrupts complex formation with VRK1.

### Design of a B12 marker rescue assay to assess B12 function.

**FIG 8:**
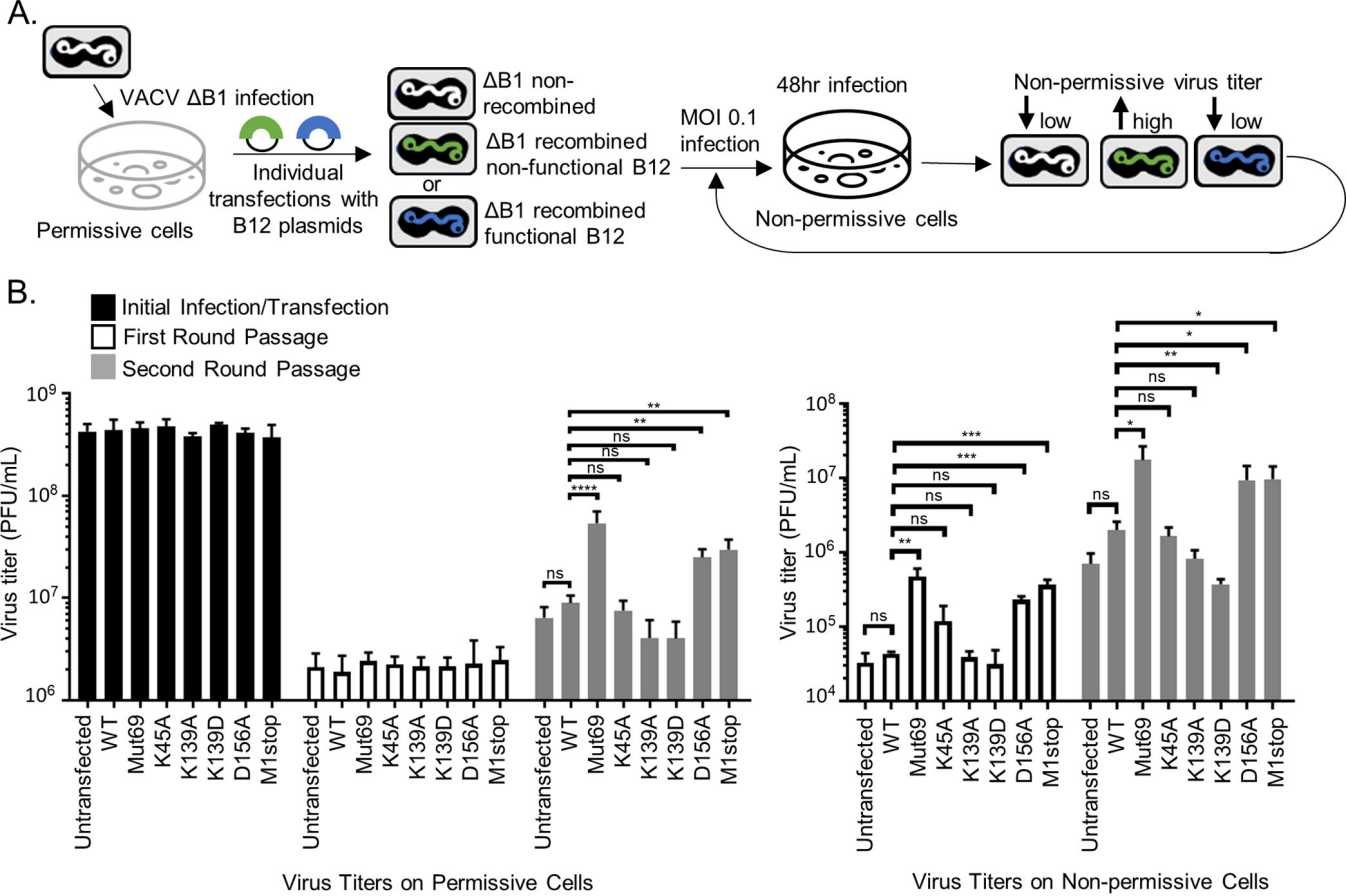
Design of a B12 marker rescue assay to assess B12 function. (A) Schematic of marker rescue assay design workflow. Briefly, ΔB1 virus (MOI = 0.1) was used to infect CV1-3×FLAGB1, and then 2 μg of the appropriate linearized plasmid DNA was transfected at 3 hpi. At 48 hpi, the virus was harvested and subsequently titrated on CV1-3×FLAGB1 (permissive) cells to determine the total amount of virus harvested, as well as on CV1 (nonpermissive, lacking B1 complementation) cells. These initial infection/transfection recombinant viruses were then infected on CV1 cells at an MOI of 0.1. Viruses were harvested at 48 hpi and titrated. The first-round passage viruses were then used to infect CV1 cells at an MOI of 0.1 for 48 h for the second-round passage, with harvesting and titration as described above. (B) Virus titers from indicated recombinant viruses performed on CV1-3×FLAGB1 (permissive cells on left) or CV1 (nonpermissive cells on right). *, *P* < 0.05; **, *P* < 0.01; ***, *P* < 0.001.

To assess the impact of B12 point mutations on its function during infection, we utilized the above-described B12 mutants in a marker rescue assay of the ΔB1 virus. We hypothesized that ΔB1 viruses that undergo homologous recombination to replace the endogenous *B12R* gene with a nonfunctional form of *B12R* would grow to higher titers on non-permissive cells in comparison to other ΔB1 viruses repressed by functional B12 (Fig. 8A). Plasmids were designed to include 150-bp sequences flanking the *B12R* gene containing desired point mutations at K45, K139, or D156. Other mutants include a previously known nonfunctional mutant of B12 that encodes for a premature stop codon at amino acid 69 (Mut69) (6), and a *B12R* gene in which the codon of the initiating methionine was altered (M1stop). The Mut69 and M1stop constructs were used as positive controls for enhanced ΔB1 virus growth when the *B12R* locus is recombined with a nonfunctional or nonexpressed B12. The initial infection and transfection of linearized plasmid DNA was performed in permissive (B1-complementing) cells to allow for optimal DNA replication and recombination to occur (Fig. 8A). At 48 hpi, virus was harvested and subsequently titrated on both permissive cells, to determine the total amount of virus harvested, as well as CV1 cells lacking B1-complementation, to determine the fraction of the total amount of virus harvested that could grow on non-permissive cells (Fig. 8A). Subsequent rounds of passage (at a multiplicity of infection [MOI] of 0.1 with 48-hpi harvests) took place on nonpermissive cells to allow for selective growth of ΔB1 viruses recombined with a nonfunctional form of B12 (Fig. 8A).

Under these conditions, similar amounts of total ΔB1 virus were harvested for each transfection group after the initial infection and plasmid transfection (Fig. 8B, left, black bars). After the first passage, the total amount of harvested virus was also similar among all transfection groups (Fig. 8B, left, white bars). Compared to the ΔB1 virus titer with WT-B12 transfection, only the B12-Mut69, B12-D156A, and B12-M1stop transfections resulted in significantly higher virus titers in non-permissive cells (Fig. 8B, right, white bars), increasing virus titers 11-, 5-, and 8-fold, respectively. The B12-K45A transfection also resulted in increased ΔB1 virus titers in noncomplementing cells by 3-fold but did not reach statistical significance. Next, a second passage of these viral stocks was performed. Notably, the total virus titers after the second round of passage revealed significantly higher titers for B12-Mut69 (6-fold), B12-D156A (3-fold), and B12-M1stop (3-fold) recombinant viruses compared to WT-B12 (Fig. 8B, left, gray bars). This is consistent with the higher titers seen for these recombinant viruses on non-permissive cells after the first passage (Fig. 8B, right, white bars) and affirms the increased ability of these mutant viruses to grow under nonpermissive conditions. In addition, the second passage recombinant viruses containing B12-Mut69, B12-D156A, and B12-M1stop B12 also exhibited higher virus titers in non-complementing CV1 cells compared to WT B12 transfection (Fig. 8B, right, gray bars), increasing virus titers 9-, 5-, and 5-fold, respectively. In contrast to the first-round titers, the B12-K45A recombinant virus in the second passage did not result in higher virus titers compared to WT-B12 in either permissive or non-permissive cells. In conclusion, these B12 marker rescue assays indicate that the D156A mutation was comparable to the positive-control Mut69 and M1stop mutations in rescuing ΔB1 virus in noncomplementing cells, strongly suggesting that this point mutation is sufficient to render B12 nonfunctional as a repressor of ΔB1 virus fitness.

### The D156A and K45A mutants are less repressive to ΔB1ΔB12 virus.

**FIG 9:**
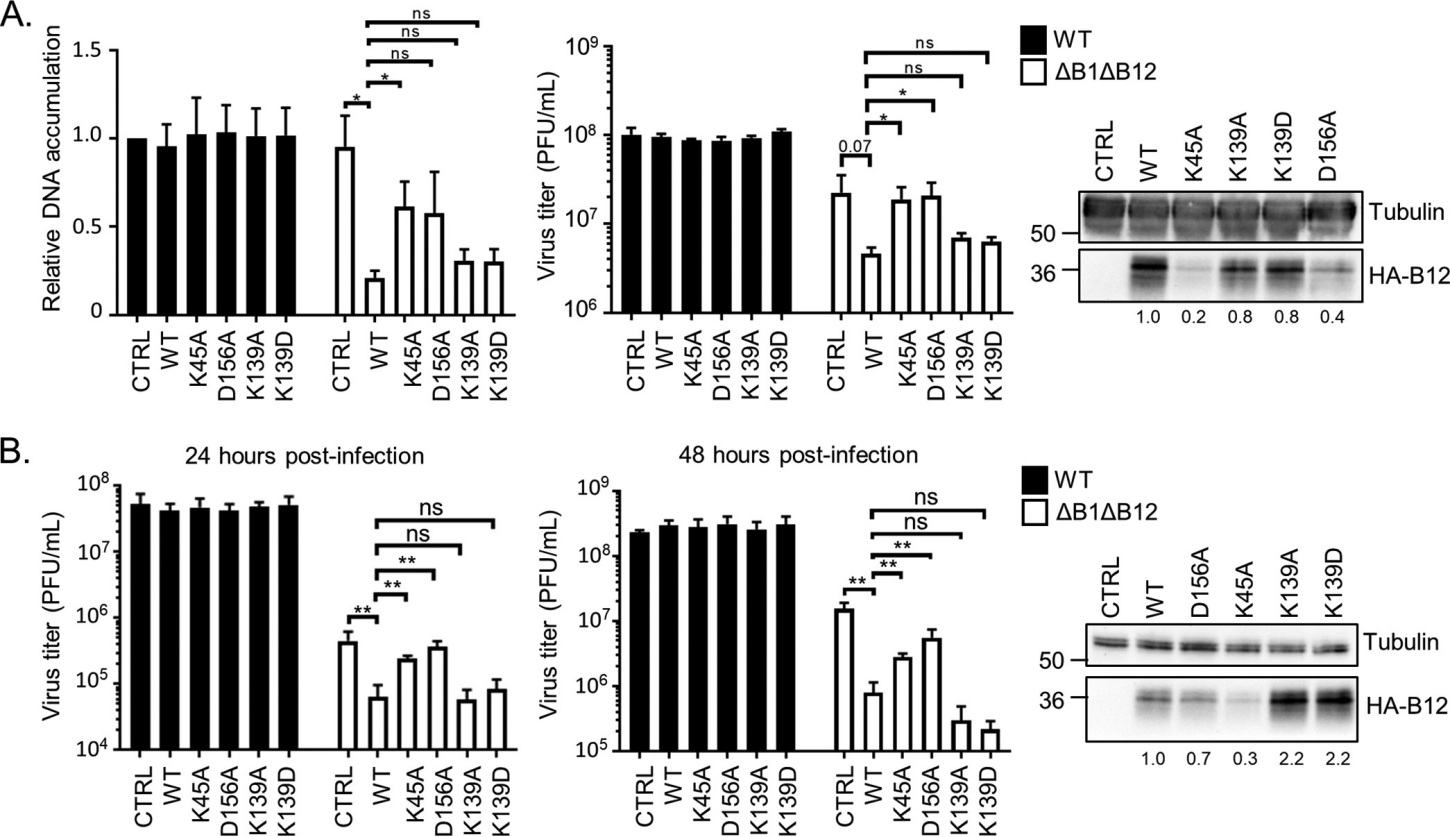
The D156A and K45A mutants are less repressive to ΔB1ΔB12 virus. (A) Viral DNA accumulation at 24 hpi based on qPCR (left) and virus titers (middle) from CV1 cells transduced with empty-vector (CTRL)-, HA-B12 (WT)-, HA-B12 (K45A)-, HA-B12 (D156A)-, HA-B12 (K139A)-, or HA-B12 (K139D)-expressing lentiviruses and infected with WT or ΔB1ΔB12 virus (MOI = 3). *, *P* < 0.05. (Right) Immunoblot analyses of whole-cell lysates used for virus infections. (B, left) Virus titers at 24 hpi (left) and at 48 hpi (middle) from CV1 cells transduced with diluted empty vector (CTRL)-, HA-B12 (WT)-, HA-B12 (K45A)-, HA-B12 (D156A)-, HA-B12 (K139A)-, or HA-B12 (K139D)-expressing lentiviruses and infected with WT or ΔB1ΔB12 virus (MOI = 0.1). **, *P* < 0.01. (Right) Immunoblot analyses of whole-cell lysates used for virus infections.

To complement our B12 marker rescue assay with cell-based expression of these B12 mutants, we next assessed the DNA replication and virus titers of WT and ΔB1ΔB12 virus on CV1 cells stably expressing HA-tagged WT-B12 or mutant B12 (Fig. 9A). We hypothesized that ΔB1ΔB12 virus would exhibit decreased DNA replication and virus titers in cells expressing functional B12, mimicking the decreased replication conditions of ΔB1 virus expressing WT B12 (6, 48). Following WT VACV infection, DNA replication (Fig. 9A, left, black bars) and virus titers (Fig. 9A, right, black bars) at 24 hpi were unaffected by stable expression of HA-B12 WT or mutants. Compared to control CV1 cells, DNA replication of ΔB1ΔB12 virus was significantly inhibited (4.5-fold) by stable expression of HA-B12-WT (Fig. 9A, left). Stable expression of HA-B12-K139A or K139D repressed ΔB1ΔB12 DNA replication in a similar way to HA-B12-WT, while DNA replication of ΔB1ΔB12 virus appeared more modestly reduced in cells expressing HA-B12-K45A or HA-B12-D156A compared to HA-B12-WT (Fig. 9A, left). The trends for ΔB1ΔB12 virus titers were similar to those seen with DNA replication, with stable expression of HA-B12-K45A or D156A significantly increasing ΔB1ΔB12 virus titers (4.4- and 4.5-fold, respectively), compared to HA-B12-WT (Fig. 9A, right). In addition, stable expression of HA-B12-K139A or K139D repressed ΔB1ΔB12 virus titers in a similar way as HA-B12-WT.

It remains possible that the HA-B12-K45A and HA-B12-D156A mutants are less repressive toward ΔB1ΔB12 replication in part due to their decreased abundance. To address this, CV1 cells were generated that expressed more comparable amounts of HA-B12-D156A, HA-B12-K45A, and HA-B12-WT by altering the amount of lentivirus used in transductions (Fig. 9B). Virus titers of WT and ΔB1ΔB12 on these cells at 24 and 48 hpi were then measured. Similar to Fig. 9A, WT virus titers at 24 hpi (Fig. 9B, left) and 48 hpi (Fig. 9B, right) were unaffected by the stable expression of HA-B12 WT or mutants. However, ΔB1ΔB12 virus titers at these time points were significantly decreased (7-fold at 24 hpi and 20-fold at 48 hpi) by WT HA-B12 expression compared to control cells and were significantly increased in cells expressing HA-B12-K45A (4-fold at 24 hpi and 3.5-fold at 48 hpi) or HA-B12-D156A (6-fold at 24 hpi and 7-fold at 48 hpi) compared to HA-B12-WT (Fig. 9B). In comparison, expression of HA-B12-K139A or HA-B12-K139D resulted in repression of ΔB1ΔB12 virus titers in a similar way as HA-B12-WT at both 24 and 48 hpi (Fig. 9B). Taken together, these results demonstrate that, unlike WT B12, B12-K139A, or B12-K139D, the ectopic expression of B12-K45A or D156A mutants are unable to repress ΔB1ΔB12 virus.

### BAF phosphorylation, cellular localization, and solubility is influenced by B12.

**FIG 10:**
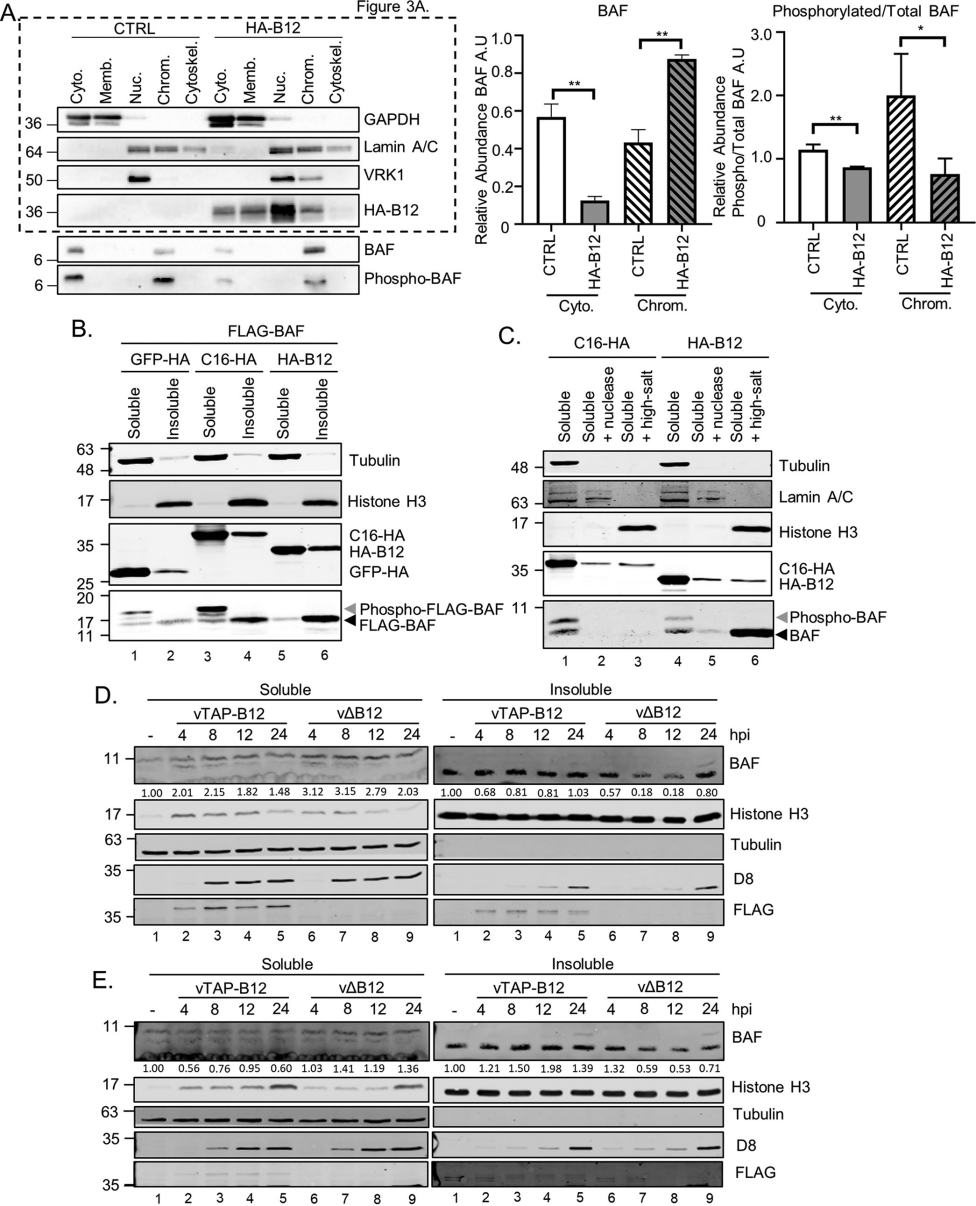
BAF phosphorylation, cellular localization, and solubility is influenced by B12. (A, left) Immunoblot analyses of subcellular fractions from CV1 cells transduced with empty vector (CTRL)- or HA-B12-expressing lentiviruses. (Right) Quantitation of GAPDH-standardized BAF (middle) or phosphorylated BAF relative to total BAF (right) abundance in each fraction, with each cell line total abundance set to 1. The results from three independent experiments used for quantitation and a representative immunoblot are shown (*, *P* < 0.05; **, *P* < 0.01). (B) Immunoblot analyses of whole-cell lysates from HEK293T cells transfected with 1.5 μg of plasmids expressing FLAG-tagged BAF in combination with HA-tagged GFP, C16, or B12 and harvested at 24 h posttransfection. (C) Immunoblot analyses of indicated detergent treatment fractions from HEK293T cells transfected with 1.5 μg of plasmids expressing HA-tagged C16 or B12. (D) Immunoblot analyses of fractions from HEK293T cells infected with vTAP-B12 or vΔB12 viruses (MOI = 5) and harvested at the indicated times postinfection. (E) Immunoblot analyses of fractions from HeLa cells infected with vTAP-B12 or vΔB12 virus (MOI = 5) and harvested at the indicated times postinfection. For quantitation in both panels D and E, the signal intensities in the BAF immunoblots of soluble and insoluble fractions were normalized to the respective noninfected controls.

Our previous study indicated that B12 can reduce VRK1-mediated phosphorylation of the DNA cross-bridging protein BAF (11). However, how this impacts the cellular properties of BAF remains incompletely understood. To better characterize the downstream consequences of B12 expression on BAF, we examined its location by subcellular fractionation. This experiment was performed in conjunction with analyses already shown in Fig. 3A, which are included here as well for reference. Immunoblot analysis of total BAF revealed a clear alteration in the partitioning of BAF upon B12 expression (Fig. 10A). Specifically, while total BAF is present at almost equal amounts in cytoplasmic and nuclear fractions in control cells, BAF is almost 90% chromatin-associated in cells expressing B12 (immunoblots are shown in Fig. 10A left and quantified on the right). Furthermore, immunoblotting with a phosphospecific BAF antibody indicate that the amount of phosphorylated BAF was also decreased in both the cytoplasmic and chromatin-associated fractions upon B12 expression (Fig. 10A).

To further study the impact of B12 on BAF cellular localization in comparison to another viral protein, we characterized the solubility of FLAG-tagged BAF in the presence of C16, another VACV protein found in the cytoplasm and nucleus (49, 50). Transient expression of HA-B12 in HEK293T cells resulted in increased detection of FLAG-BAF in the insoluble fraction compared to the higher detection of FLAG-BAF in the soluble fraction of GFP-HA- and C16-HA-expressing cells (Fig. 10B, compare lanes 1, 3, and 5). In addition, the higher-molecular-weight forms detected for FLAG-BAF in the GFP-HA and C16-HA soluble samples were completely absent with HA-B12 expression (Fig. 10B). These slower migrating forms of BAF have been characterized as phosphorylated forms of BAF (24), therefore suggesting that HA-B12 also decreases the phosphorylation of BAF in this assay. Additional characterization of the solubility of endogenous BAF in HEK293T cells following sequential nuclease and high-salt treatments revealed that only HA-B12 resulted in BAF repartitioning to the high-salt fraction (Fig. 10C, lane 6), with BAF detected in C16-HA-expressing HEK293T cells in only the soluble conditions (Fig. 10C, lanes 1 and 7). These data indicate that in the absence of infection, B12 influences the phosphorylation state of BAF and simultaneously alters its subcellular localization and/or solubility properties.

To investigate how B12 impacts BAF during infection, the solubility of BAF in HEK293T (Fig. 10D) and HeLa (Fig. 10E) cells was examined during infection with either B12-deficient (vΔB12) virus or the revertant virus expressing N-terminal TAP-tagged B12 from its natural locus (vTAP-B12). During infection, the absence of B12 resulted in less BAF detected in the insoluble fractions at 8 and 12 hpi (Fig. 10D, right, lanes 7 and 8, and Fig. 10E, right, lanes 7 and 8). This correlated with increased detection of BAF in the soluble fractions during vΔB12 infection (Fig. 10D, left, lanes 7 to 9, and Fig. 10E, left, lanes 7 to 9). Taken together, these results demonstrate that B12 influences the subcellular localization, solubility, and phosphorylation state of BAF.

### B12 redirects BAF intracellular localization.

**FIG 11:**
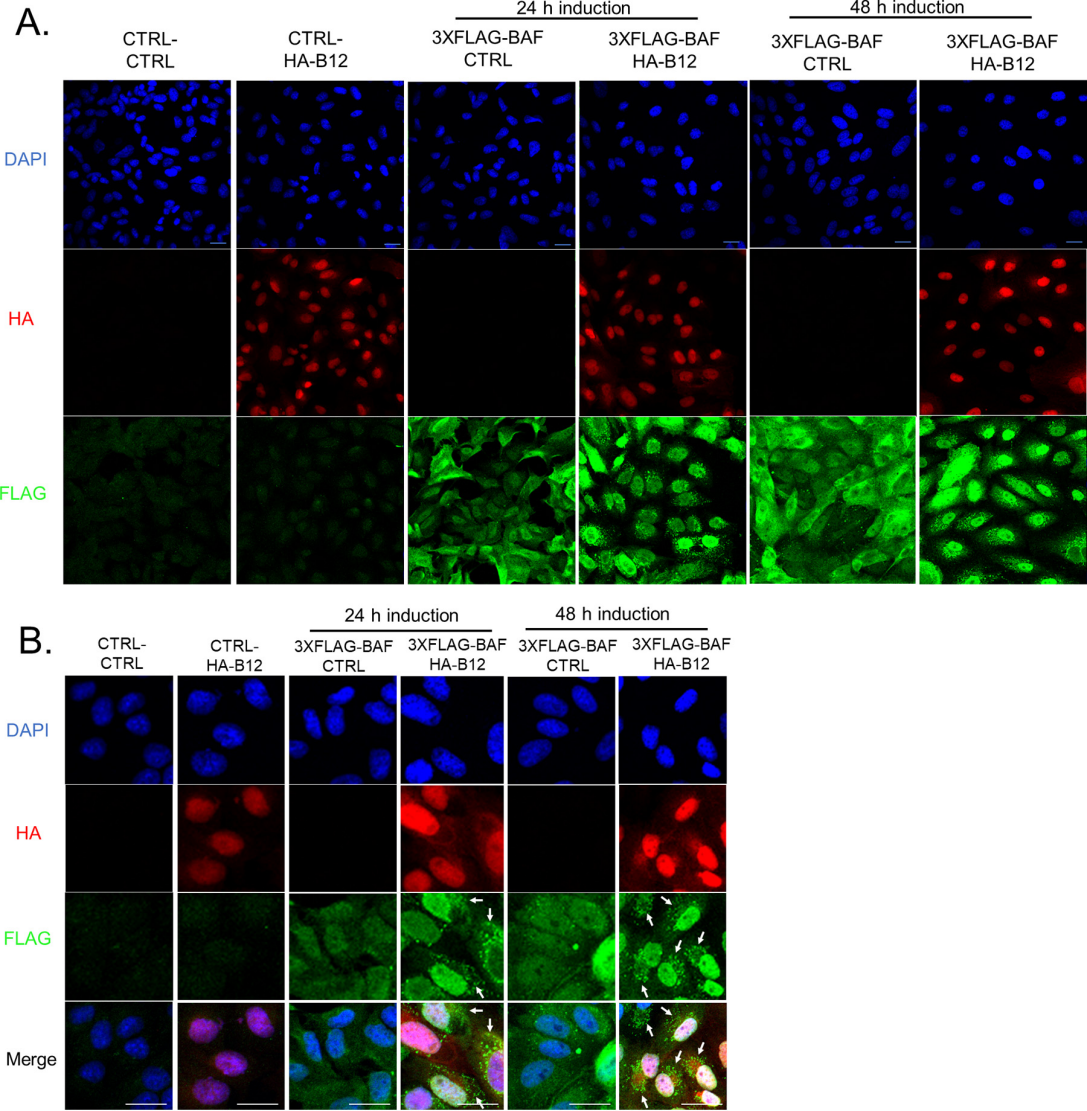
B12 redirects BAF intracellular localization. (A and B) Immunofluorescence of CV1 cells transduced with empty-vector (CTRL)-, 3×FLAG-BAF-, and HA-B12-expressing lentiviruses. DAPI (nuclei), HA (B12) and FLAG (BAF) were detected. Scale bar, 20 μm.

To expand and confirm the above observations, we next examined the localization of 3×FLAG-BAF in B12-expressing cells by immunofluorescence microscopy. Stable expression of 3×FLAG-BAF in control CV1 cells resulted in relatively equal detection of both nuclear and cytoplasmic BAF, in agreement with previous studies (51) (Fig. 11A). Interestingly, the stable expression of HA-B12 with 3×FLAG-BAF for 24 or 48 h resulted in the decreased detection of BAF in the cytoplasm and more distinct detection within the nucleus (Fig. 11A). The predominantly nuclear localization of HA-B12 was unaffected by the expression of 3×FLAG-BAF (Fig. 11A). Closer examination of CV1 cells coexpressing 3×FLAG-BAF and HA-B12 for 24 h more clearly demonstrated the increase in nuclear 3×FLAG-BAF intensity and revealed small clusters of condensed cytoplasmic BAF-positive foci which became more abundant after 48 h of HA-B12 expression (Fig. 11B). In conclusion, these results suggest that B12 expression has a profound impact on the intracellular localization of BAF.

### Interferon production downstream of DNA sensing is increased by B12.

**FIG 12:**
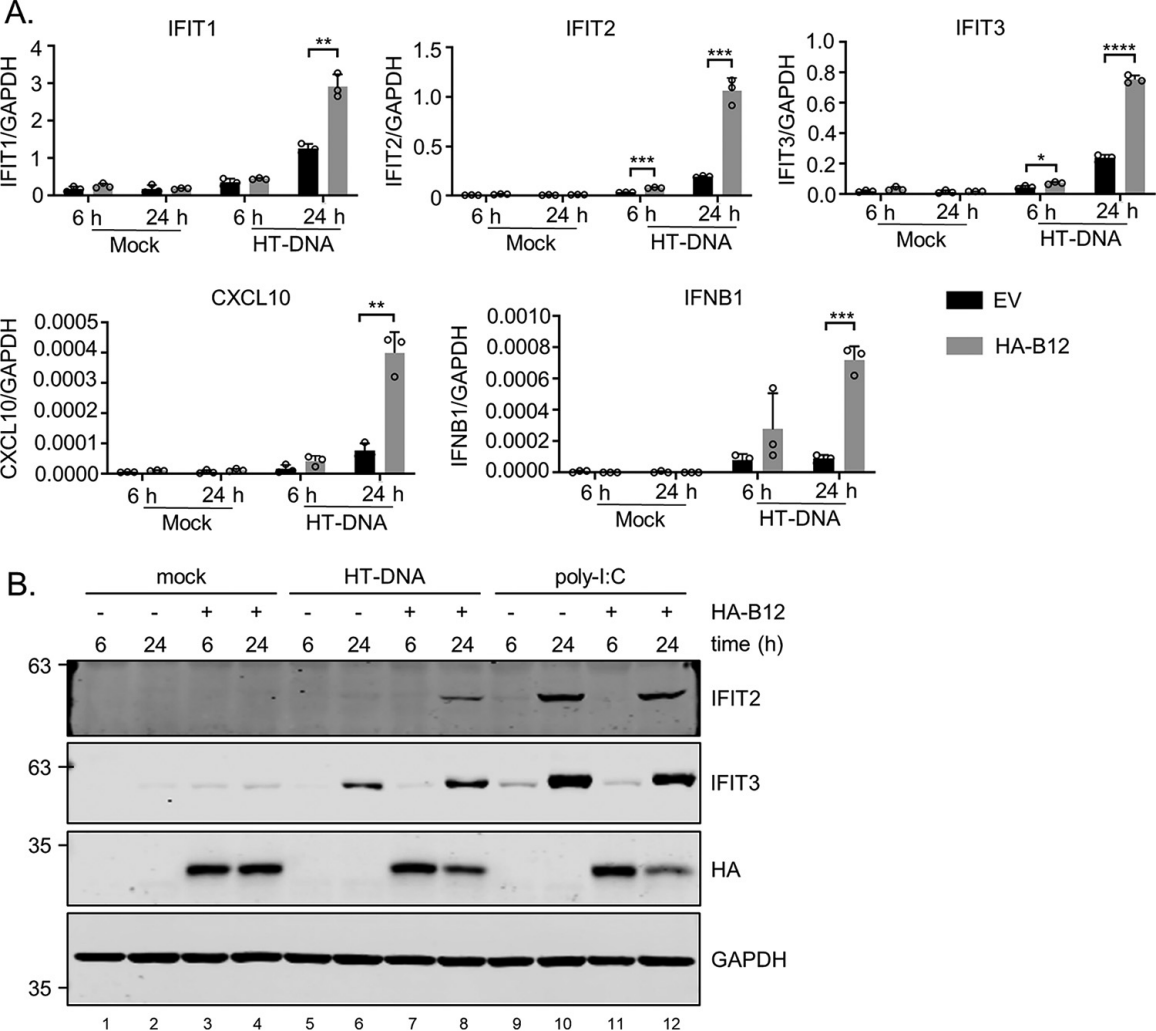
DNA-induced IFN response is increased in the presence of B12. (A) RT-qPCR of *IFIT1*, *IFIT2*, *IFIT3*, *CXCL10*, and *IFNB1* mRNA expression from HFF-TERT cells transduced with empty vector (EV)- or HA-B12-expressing lentiviruses and transfected with mock or HT DNA (1 μg/mL) for 6 or 24 h. Three technical replicates (cells from three individual wells) are shown for each sample (**, *P* < 0.01; ***, *P* < 0.001; ****, *P* < 0.0001). (B) Immunoblot analyses of whole-cell lysates from HFF-TERT cells transduced with empty vector (EV)- or HA-B12-expressing lentiviruses and transfected with mock, HT-DNA (1 μg/mL) or high-molecular-weight poly(I·C) (0.5 μg/mL) for 6 or 24 h.

Given the characterized role of other poxvirus proteins such as N2 and C6 in blocking the production of interferon (IFN) or IFN-induced signaling (52–55), we posited that B12 may be capable of a similar function. The impact of B12 on BAF led us to investigate dsDNA-responsive signaling previously reported to be affected by BAF (39, 40). Telomerase reverse transcriptase (TERT)-immortalized human foreskin fibroblast (HFF-TERT) cells were transduced with empty vector (EV)- or HA-B12-expressing lentiviruses for stable, inducible expression of HA-B12. After 24 h of doxycycline induction, cells were mock transfected or transfected with herring testis (HT) DNA for 6 or 24 h, followed by quantification of mRNA expression of IFN-β (*IFNB1*), and IFN-stimulated genes (ISGs) (Fig. 12A). Upon DNA transfection, cells transduced with HA-B12 expressed higher levels of *IFNB1* and *CXCL10* and downstream ISGs *IFIT1*, *IFIT2*, and *IFIT3* and *CXCL10* mRNAs compared to control cells at 24 h (Fig. 12A). For the ISGs *IFIT2* and *IFIT3*, higher mRNA levels were also seen at 6 h poststimulation with HT-DNA in the presence of B12 (Fig. 12A).

Next, we sought to verify these results at the protein level and investigated if stimulation by the double-stranded RNA analog poly(I:C) also increased ISG product expression in the presence of HA-B12. Undetectable to low levels of both IFIT2 and IFIT3 were detected in both mock-transfected control and HA-B12-expressing cells (Fig. 12B, lanes 1 to 4). Treatment with HT-DNA for 24 h induced increased IFIT2 and IFIT3 protein levels in HA-B12-expressing cells to higher levels compared to control cells (Fig. 12B, compare lanes 6 and 8). However, poly(I:C) stimulation for 24 h did not induce increased protein expression of IFIT2 and IFIT3 in HA-B12-expressing compared to control cells (Fig. 12B, compare lanes 10 and 12). This indicates that increased ISG induction in the presence of HA-B12 appears to be specific to DNA stimulation. Together, these data demonstrate that cells expressing B12 exhibit increased sensitivity to immunostimulatory DNA and suggest that this viral pseudokinase promotes innate immune signaling.

## DISCUSSION

**FIG 13:**
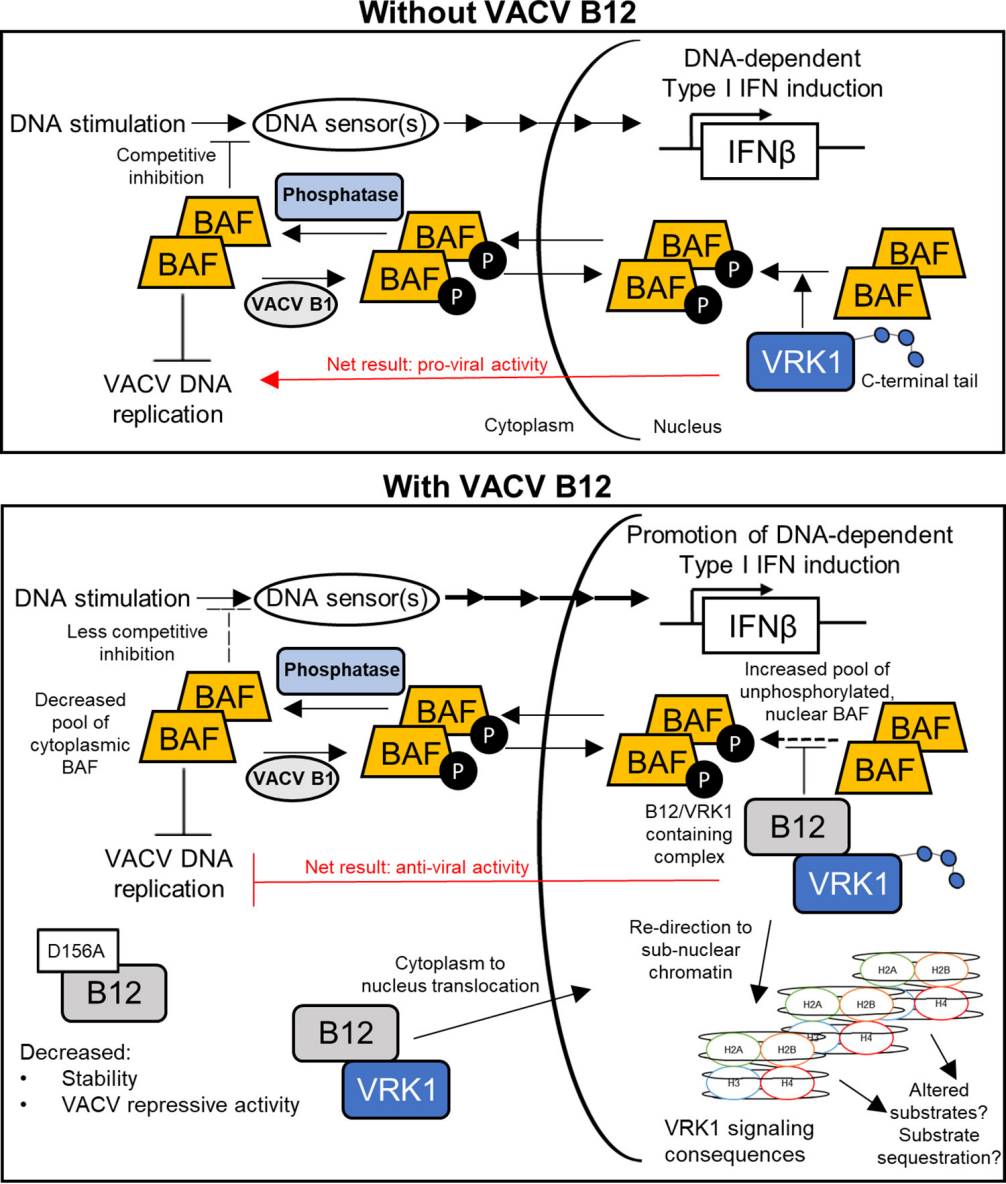
Summary working model. This study details several consequences of B12 regulation of VRK1, including the upregulation of DNA-dependent type I IFN expression. Data here and in previous studies inform this working model. (Top) In the absence of B12, VRK1 phosphorylates BAF in the nucleus, a property that is linked to its VACV proviral function (11, 71). BAF shuttling between the nucleus and the cytoplasm depends on its phosphorylation state, with unphosphorylated BAF preferentially found in the nucleus (38, 51). Unphosphorylated BAF in the cytoplasm has high affinity for DNA (83, 101), which contributes to repression of VACV DNA replication (36, 48), and dampens the sensing of cytosolic dsDNA and the induction of type I IFN (39, 40). (Bottom) In the presence of B12, B12 complex formation with VRK1 results in their redirection to chromatin. Downstream consequences of VRK1 relocalization remain to be completely determined but correlate with reduced VRK1-dependent BAF phosphorylation, resulting in the retention of unphosphorylated BAF in the nucleus. Our model predicts that this decrease in unphosphorylated BAF in the cytoplasm results in increased sensing of immunostimulatory DNA in the cytoplasm by DNA sensors, explaining the increase in innate immune responses in the presence of B12. We also characterized a panel of VRK1 and B12 mutants, discovering a B12-D156A mutation that disrupts complex formation with VRK1 and augments its repressive VACV DNA replication activity. These studies provide evidence of multiple levels of mutual regulation between the VRK1 and B12 proteins.

While the critical role of protein pseudokinases in governing molecular signal transduction is becoming increasingly apparent, the contribution of pseudokinases during viral infection remains poorly understood. The VACV *B12R* gene encodes a pseudokinase capable of acting as a potent inhibitor of VACV DNA replication in the absence of VACV B1 regulation (6, 11, 48, 56). Previous studies indicate that B12 impacts the poxviral life cycle via association with VRK1, a cellular kinase with which B12 copurifies and may redirect to the nucleus during infection (11). Positing not only that B12 regulates VRK1 but also that VRK1 may modulate B12, we pursued an interconnected series of structure/function studies of these two proteins alone and in combination. The studies here provide compelling evidence of mutual regulation of both proteins at multiple levels and provides new insights into how a B12/VRK1 complex repurposes cellular signaling during poxviral infection. To that point, we found that B12 expression concomitantly triggered BAF relocalization to the nucleus and increased sensitivity to cytoplasmic DNA sensing. Assembly of these data has led to our current working model that the functional interaction between B12 and VRK1 increases BAF retention in the nucleus, resulting in unanticipated consequences to the innate immune response downstream of DNA sensing (Fig. 13).

To begin our studies, we first investigated the properties of B12 and VRK1 when coexpressed and found that VRK1 stabilizes B12. Next, we used multiple complementary assays examining the subcellular fractionation and localization of the two proteins in uninfected and infected cells and found that VRK1 and B12 each modulate the intracellular localization of the other. First, consistent with prior research (18), we found detectable amounts of VRK1 in insoluble fractions when it was expressed alone. Indeed, both human VRK1 and its other evolutionary homologs have been demonstrated to be histone kinases (12, 18, 57, 58). However, the proportion of VRK1 observed in those salt-resistant fractions increased greatly in a B12-dependent manner. Moreover, the fractionation profile of the two proteins overlaps considerably with each other as well as with histones, suggesting that B12 and VRK1 are associated with chromatin. Second, prior reports have also demonstrated that VRK1 adopts a diffuse localization during mitosis, as observed using immunofluorescence microscopy (13). We affirmed these results with GFP-VRK1 in our cell models as well; however, in cells transduced to express B12, GFP-VRK1 is instead colocalized with condensed mitotic chromatin. Overall, these data suggest that assembly of a B12/VRK1-containing complex promotes the ability of both factors to interact with other nuclear proteins, likely those enriched in chromatin. Since VRK1 is known to regulate mitosis-specific events (59, 60), it is worth considering that the properties of a B12/VRK1 complex might parallel with those of other mitotic kinases controlled by both regulatory partners and chromatin localization (61–69). Indeed, based in part on precedents from the mitotic kinase Aurora kinase B, it is tempting to speculate that B12 can modulate VRK1’s activity in regulating cell division (59, 60) via BAF and other potential substrates. Interestingly, a recent study demonstrated that VACV does modulate host cell proliferation in a manner dependent on B1 and B12 (70).

Employing a series of VRK1 mutants, we demonstrated that the region necessary for coprecipitation of VRK1 and B12 lies outside VRK1’s C-terminal tail, suggesting that B12/VRK1 complex formation depends upon the VRK1 catalytic domain. In addition, since the removal of both NLS segments relocates VRK1 to the cytoplasm, nuclear localization and additional nuclear cellular proteins also appear unnecessary for B12/VRK1 complex formation. Interestingly, we also observed that VACV infection was sufficient to drive a portion of cytoplasmic VRK1 into the nucleus along with B12. These data highlight the interconnected properties of the B12/VRK1 complex for nuclear-mediated events. Additional investigation into the functional properties of the cytoplasmic VRK1 mutants demonstrated that they were unperturbed in their proviral VACV activity. These results affirm and extend prior studies demonstrating that VRK1 expressed in the cytoplasm from the VACV genome can enhance viral replication (71). Together with prior evidence that VRK1 mediates proviral signaling via BAF and other yet-to-be-identified downstream substrates (11), our data suggest that these substrates are likely present in both the nucleus and cytoplasm in infected cells.

In conjunction with VRK1 mutagenesis efforts, we designed and screened for B12 loss-of-function mutations as measured by both VRK1 coimmunoprecipitation and repression of ΔB1 VACV replication. To functionally characterize these B12 mutants, we utilized both a marker rescue and cell-transgene complementation assay. The B12 marker rescue assay relies upon greater fitness and higher virus titers demonstrated for ΔB1 recombinant viruses that contain a nonfunctional *B12R* gene compared to those containing a functional *B12R* gene (6). Interestingly, only the recombinant viruses derived from B12 D156A transfection grew to significantly higher titers compared to the WT-B12 ΔB1 recombinant viruses. Since these titers were similar to titers of the nonfunctional Mut69-B12 recombinant virus, we surmise that the D156A mutation renders B12 inactive in this assay. In comparison, although the K45A mutant renders B12 less abundant, it was unable to enhance viral yield to the same degree as M1stop-B12 during marker rescue, indicating that even low levels of functional B12 are sufficient to inhibit ΔB1 recombinant virus growth in this assay. The cellular transgene complementation assay provides additional insights into the function of B12 mutants during repression of the ΔB1ΔB12 virus. In this system, a lack of repression of the ΔB1ΔB12 virus was observed with both the B12 D156A and K45A mutants, arguing that in some contexts either mutation is sufficient to cause a loss of function. Overall, the combination of both the marker rescue assay and cellular transgene complementation assay provide evidence that the D156A mutant is a nonrepressive B12 variant, strongly indicating that complex formation between VRK1 and B12 is connected to B12 function during ΔB1 infection.

These data are consistent with a working model in which B12 may contribute to allosteric regulation of VRK1 activity, perhaps via direct interaction or via other unknown components of a B12/VRK1-containing complex. Indeed, multiple precedents exist for pseudokinases acting as regulators of active enzymes, including active protein kinases (1, 4, 72, 73). One well-characterized example of this mode of regulation is the JAK2 pseudokinase domain, which directly binds and negatively regulates the kinase domain of all JAK family members (74, 75). In other cases, pseudokinases can act as scaffolds, facilitating contact between kinases and their substrates or tethering the enzyme to larger complexes (4, 76). Our finding that the sites involved in copurification of B12 and VRK1 likely lies within the catalytic domain of VRK1 supports the hypothesis that B12 may be directly regulating the VRK1 catalytic domain. Regulation of some protein kinases occurs via regulation of its conformational switch between a closed, catalytically productive conformation and an open, unproductive conformation (77–79). However, current structural studies of VRK1 indicate it is locked in a closed conformation (46, 80), indicating that other mechanisms of regulation may be involved. Thus, B12 regulation of VRK1 may either result from a novel conformational adjustment or may instead result from VRK1 relocalization within the cell. This relocalization may redirect VRK1 away from some substrates, such as BAF. If true, it is tempting to speculate that B12 may also be redirecting VRK1 toward other substrates that await future identification.

Our previous study indicated that B12 engages with VRK1-mediated signaling during VACV infection and is sufficient to prevent VRK1-mediated phosphorylation of BAF (11). Here, we further investigated whether this B12-mediated consequence required the action of other viral proteins, and how this was impacting the intracellular behavior of BAF. Using biochemical fractionation and immunofluorescence, we found that B12 alone is sufficient to decrease BAF phosphorylation and shift BAF localization from the cytoplasm to the nucleus. The correlation between BAF phosphorylation state and its subcellular location is consistent with prior studies that examined the predominant nuclear localization of unphosphorylated BAF (51). In addition, the shift in BAF to the nucleus and an increase in dephosphorylated BAF also correlated with a decrease in the solubility of BAF and was demonstrated with either B12 expression alone or in the context of VACV infection. Intriguingly, after longer periods of B12 expression, BAF not only becomes predominantly nuclear, but also relocalizes to cytoplasmic puncta. Since unphosphorylated BAF has been shown to bind and condense DNA in a sequence-independent manner (81–83), one possibility is that extrachromosomal DNA triggers formation of these puncta. If this is the case, the mechanism for how such DNA enters the cytoplasm remains to be determined. It is interesting to speculate that loss of nuclear envelope integrity and/or dysregulated mitosis may also result from B12 expression and explain these puncta, given that regulation of BAF phosphorylation state is heavily implicated in these processes (38).

In addition to BAF regulation, we examined whether B12 expression affected other potentially connected antiviral pathways. Interestingly, we found that levels of IFN-β, CXCL10 and ISGs mRNAs stimulated by exogenous DNA were higher in B12-expressing cells compared to control cells, indicating that the presence of B12 alone can promote the innate immune signaling downstream of cytosolic DNA sensing. This upregulation was specific to DNA, as poly(I:C) did not show the same difference between B12-expressing and control cells. The ability of B12 to enhance DNA induced signaling contrasts with the inhibition of innate immune signaling by many other immunomodulatory VACV proteins (84). VACV blocks the sensing of cytosolic PAMPs (e.g., cytosolic dsDNA) (50, 85–87), the signaling cascades that activate IFN production (52, 53, 88, 89), type I IFN signaling (54, 90, 91), and the actions of ISGs (92, 93). However, the finding that a pathogen-encoded protein results in augmentation of innate immune signaling is not unique (94) and emphasizes the complexity of the cross-regulatory immunity networks of the host (95, 96). In line with the recent implications of BAF in dampening responses to endogenous and exogenous DNA sensing (39, 40), a plausible mechanism is that the formation of the B12/VRK1 complex leads to the reduced phosphorylation of BAF and its redistribution to the nucleus, away from the cytoplasm where VACV replicates. Consequently, the reduced pool of cytoplasmic BAF increases the cell’s sensitivity to cytosolic DNA sensing by PRRs, thereby leading to enhanced production of IFN-β and downstream ISGs. Although beyond the scope of the present study, determining the mechanism of how B12 leads to upregulation of innate immune signaling will be informative.

In summary, together with prior studies, the data presented here provide strong evidence for a functional link between B12 and VRK1 that may be remodeling VRK1 signaling in the nucleus, resulting in the dysregulation of BAF and the innate immune response and influencing other, not-yet-identified substrates of VRK1 (Fig. 13). Indeed, in addition to BAF, VRK1 modulates a complex array of transcription factors and histones, culminating in altered responses to DNA damage and cell cycle control, many of which may be impacted by B12. Thus, future inquiry of how B12 regulation of VRK1 reshapes these signaling events may yield new insights into nuclear pathways targeted by poxviruses.

## MATERIALS AND METHODS

### Chemicals and reagents.

Chemicals were obtained from Sigma-Aldrich or Fisher Scientific unless otherwise stated. Primers were obtained from Integrated DNA Technologies.

### Cell culture.

Human near-haploid fibroblast HAP1 parental and VRK1 knockout (VRK1KO) cell lines were obtained from Horizon Genomics, maintained in Iscove modified Dulbecco medium supplemented with 10% fetal bovine serum (FBS; Atlanta Biologicals) and penicillin-streptomycin, and incubated at 37°C in a 5% CO_2_ atmosphere. VRK1KO cells (catalog no. HZGHC000073c014) contain an 11-bp deletion in VRK1 exon 5. African green monkey kidney CV1 cells were purchased from Invitrogen Life Technologies. Human cervix epithelial adenocarcinoma HeLa cells, human lung epithelial carcinoma A549 cells, human embryo kidney 293T (HEK293T) cells, monkey kidney BS-C-1 cells, and rabbit kidney RK13 cells were obtained from the American Type Culture Collection (ATCC). CV1, BS-C-1, HeLa, and HEK293T cell lines were maintained in the Dulbecco modified Eagle medium (DMEM) supplemented with 10% FBS, 100 U/mL penicillin (Life Technologies), and 100 μg/mL streptomycin (Life Technologies) in 5% CO_2_ at 37°C. A549 cells were maintained in DMEM/Ham F-12 nutrient mixture (1:1) medium supplemented with 10% FBS and penicillin-streptomycin under conditions identical to those described stated above. RK13 cells were maintained in minimum essential medium (MEM; Gibco) supplemented with 10% FBS and penicillin-streptomycin under conditions identical to those stated above. Telomerase reverse transcriptase (TERT)-immortalized human foreskin fibroblast (HFF-TERT) cells were kindly provided by Michael P. Weekes (University of Cambridge, UK) and were maintained in DMEM supplemented with 10% FBS and penicillin-streptomycin.

### Viruses and viral infections.

Wild-type VACV strain Western Reserve (WR), B1-deleted virus (ΔB1) (48) WRHAB12 virus (6), ΔB1ΔB12 virus (described below), MutB12 (described below), WTeHAB12 (described below), vTAP-B12 (described below), and vΔB12 (8) were used for VACV infections. The WT, ΔB1, WRHAB12, ΔB1ΔB12, MutB12, and WTeHAB12 viruses were expanded on CV1, or CV1-3×FLAGB1 cells, purified by ultracentrifugation on a 36% (wt/vol) sucrose cushion, and stored at −80°C in 1 mM Tris (pH 9).

vTAP-B12 and vΔB12 virus stocks were prepared in RK13 cells. Cells grown to confluence in T-175 flasks were infected at 0.01 PFU/cell until a complete cytopathic effect was visible. The cells were harvested by centrifugation, suspended in a small volume of DMEM supplemented with 2% FBS, and submitted to multiple cycles of freezing/thawing and sonication to lyse the cells and disrupt aggregates of virus particles and cellular debris. These crude virus stocks were used for all the experiments. The viral titers were determined by plaque assay on BS-C-1 cells.

For VACV DNA accumulation and titer determination, confluent monolayers of cells were infected with WT, ΔB1, or ΔB1ΔB12, at an MOI of 3 or 0.1 at 37°C for 24 h. Cells were harvested into 400 μL of phosphate-buffered saline (PBS) and aliquoted (200 μL for viral DNA accumulation and 200 μL for viral titer determination) prior to downstream assays. For viral yield assays, following cell harvest, cells were collected by centrifugation and resuspended in 200 μL of 10 mM Tris (pH 9). Virus samples were freeze-thaw lysed three times and infectivity was titrated on CV1 or CV1-3×FLAGB1 cells at 37°C. For plaque assays, confluent monolayers of transduced CV1 cells in 6-well plates were infected with WT or ΔB1 at 300 PFU at 37°C for 72 h. For protein immunoprecipitations, immunoblotting, and fractionation experiments, confluent monolayers of cells were infected with the indicated VACV strains at the indicated MOIs and lengths of time prior to lysate collection.

### Cloning of constitutive lentivirus expression vectors.

pHAGE-HYG-MCS (97), pHAGE-HYG-3×Flag-BAF (the plasmid was generously provided by Paula Traktman), pHAGE-HYG-3×FLAGB1 (56), and pHAGE-HYG-3×FLAGVRK1 (11) were described previously. pHAGE-HYG-3×FLAGVRK1-ΔNLS, pHAGE-HYG-3×FLAGVRK1-ΔBasic, pHAGE-HYG-3×FLAGVRK1-ΔNLSΔBasic, pHAGE-HYG-3×FlagVRK1 1-369, and pHAGE-HYG-3×FLAGVRK1 1-331, pHAGE-HYG-meGFP, and pHAGE-HYG-meGFPhVRK1 were produced through overlap PCR and cloning. All PCR primers are listed in Table 1. 3×FLAGVRK1-ΔNLS PCR product was generated by overlap PCR on 3×FLAGVRK1 using F-FLAG that contained a BamHI restriction site at the 5′ end of 3×FLAG and R-VRK1-ΔNLS that contained the A^356^AAA substitutions as well as F-VRK1-ΔNLS and R-VRK1 that contained the 3′ end of VRK1 and a BamHI restriction site. In the second step, all the PCR products from the above PCRs were combined and amplified into one PCR product with the F-FLAG and R-VRK1 primers. 3×FLAGVRK1-ΔBasic PCR product was generated by PCR on 3×FLAGVRK1 using F-FLAG and R-VRK1-ΔBasic that removed 10 amino acids from the C-terminal end of VRK1 and contained a BamHI restriction site. 3×FLAGVRK1-ΔNLSΔBasic PCR product was generated by PCR on the 3×FLAGVRK1ΔNLS PCR product using the above-described F-FLAG and R-VRK1-ΔBasic primers. 3×FLAG-VRK1 1-369 PCR product was generated by PCR on 3×FLAGVRK1 using F-NheI-FLAG that contained a NheI restriction site at the 5′ end of 3×FLAG and R-VRK1 1-369 that removed 27 amino acids from the C-terminal end of VRK1 and contained a BamHI restriction digest site. 3×FLAG-VRK1 1-331 PCR product was generated by PCR on 3×FLAGVRK1 using F-NheI-FLAG and R-VRK1 1-331 that removed 65 amino acids from the C-terminal end of VRK1 and contained a BamHI restriction digest site. meGFP (A206K mutation) PCR product was generated by overlap PCR on eGFP using F-NheI-eGFP that contained a NheI restriction site at the 5′ end of eGFP and R-A206K-eGFP that contained the A206K mutation as well as F-A206K-eGFP and R-BamHI-eGFP that contained the 3′ end of eGFP and a BamHI restriction site. In the second step, all the PCR products from the above PCRs were combined and amplified into one PCR product with the F NheI-eGFP and R BamHI-eGFP primers. meGFP-VRK1 PCR product was generated by dual overlap PCR. In the first step, meGFP plasmid was used as the template for F-NheI-eGFP and R-meGFP-VRK1 to amplify meGFP containing 25 bp encoding the N terminus of VRK1. 3×FLAGVRK1 was used as the template for F-meGFP-VRK1 and R-BamHI-VRK1 to amplify VRK1 containing 25 bp encoding the C terminus of meGFP. In the second step, all the PCR products from the above PCRs were combined and amplified into one PCR product with the F-NheI-eGFP and R-BamHI-VRK1 primers. All PCR products were digested with BamHI and/or NheI, and ligated into the multiple cloning site of BamHI- and/or NheI-digested pHAGE-HYG-MCS. Plasmids were verified by DNA sequencing.

**TABLE 1:** Oligonucleotide primers

Primer	Sequence (5′–3′)^*a*^
F-Flag	GAGA*GGATCC*GCCACC**ATGGACTACAAAGACCATGACGG** (BamHI)
R-VRK1-ΔNLS	GCTTTCTTCAATTTCTGCCGCTGCCGCCGCTGTTATTGTTTTTGC
F-VRK1-ΔNLS	GCAAAAACAATAACAGCGGCGGCAGCGGCAGAAATTGAAGAAAGC
R-VRK1	GAGA*GGATCC*TTACTTCTGGACTCTCTTTCTGGTTCTTGAACGGGTCTGT (BamHI)
R-VRK1-ΔBasic	GA*GGATCC*TTAGGTCTGTATGGCCTCCTCTGTCTGTGTGTTTGACCAT (BamHI)
F-NheI-FLAG	GAGA*GCTAGC*GCCACC**ATGGACTACAAAGAC** (NheI)
R-VRK1 1-369	TCTC*GGATCC*TTAACCAGGTTCCTTGCTTTCT (BamHI)
R-VRK1 1-331	TCTC*GGATCC*TTATATAGCTTTTAGTCCTTGCAA (BamHI)
F-NheI-eGFP	GAGA*GCTAGC***GCCACC**ATGGTGAGCAAGGG (NheI)
R-A206K-eGFP	GGGGTCTTTGCTCAGCTTGGACTGGGTGCTCAG
F-A206K-eGFP	CTGAGCACCCAGTCCAAGCTGAGCAAAGACCCC
R-BamHI-eGFP	TCTC*GGATCC*TTACTTGTACAGCTCGTCCA (BamHI)
R-meGFP-VRK1	CTTGAGCTGCTTTTACACGAGGCATCTTGTACAGCTCGTCCATGCCGAGA
F-meGFP-VRK1	TCTCGGCATGGACGAGCTGTACAAGATGCCTCGTGTAAAAGCAGCTCAAG
R-BamHI-VRK1	GAGA*GGATCC*TTACTTCTGGACTCTCTTTCTGGTTCTTGAACGGGTCTGT (BamHI)
F-HAB12	TAGATA*GCTAGC*GCCACC**ATGTATCCCTACGACG** (NheI)
R-B12-K45A	GCTCTTGTGGTCGATGGCCATCACGTAGTTGTA
F-B12-K45A	TACAACTACGTGATGGCCATCGACCACAAGAGC
R-HAB12	ATAAGT*GCTAGC*TTAGTCCTGGATGAACAGCTTCCGC (NheI)
R-B12-D156A	GTTGGTCCGGCTGTAGGCGATCAGGCTCAGCCG
F-B12-D156A	CGGCTGAGCCTGATCGCCTACAGCCGGACCAAC
R-B12-K139A	GTTTCTGGGCTCGATTGCGCCGTGGGTGAAGCC
F-B12-K139A	GGCTTCACCCACGGCGCAATCGAGCCCAGAAAC
R-B12-K139D	GTTTCTGGGCTCGATATCGCCGTGGGTGAAGCC
F-B12-K139D	GGCTTCACCCACGGCGATATCGAGCCCAGAAAC
F-EcoRI-B11	GAGT*GAATTC*TGCTTACTACTAACATGGATACAGA (EcoRI)
R-EcoRI-B13	GACT*GAATTC*TCAATACTGACGAGATTGAC (EcoRI)
R-mut69B12	CGATAGTCAAAGGATCCCCAATACAGATAT
F-mut69B12	ATATCTGTATTGGGGATCCTTTGACTATCG
R-K45A-B12	TGTGATTTGTGATCTATGGCCATTACGTAATTCTA
F-K45A-B12	TACAATTACGTAATGGCCATAGATCACAAATCACA
R-K139A-B12	CCTCGGTTCTATGGCTCCATGGGTAAAT
F-K139A-B12	ATTTACCCATGGAGCCATAGAACCGAGG
R-K139D-B12	CCTCGGTTCTATATCTCCATGGGTAAATCC
F-K139D-B12	GGATTTACCCATGGAGATATAGAACCGAGG
R-D156A-B12	GTTAGTTCTAGAATAGGCAATTAGTGAAAGACGTTT
F-D156A-B12	AAACGTCTTTCACTAATTGCCTATTCTAGAACTAAC
R-M1stop-B12	ATACTTGAAGGATTCTCAGCCTGCTATGTAATA
F-M1stop-B12	TATTACATAGCAGGCTGAGAATCCTTCAAGTAT
F-A57	GATATGGATGAGGCCAACGAAGC
R-B2	CTCAAACATAGGCAGCAGTGCTCC
F-XhoI-B11	TCTG*CTCGAG*TTGCTTACTACTAACATGGATACAG (XhoI)
R-XhoI-B12	TCTG*CTCGAG*TTCAGTATCCTTTGGGGCGA (XhoI)
F-HA	CATCATCTGGAATTGTCACTACTAAA
R-HA	ACGGCCGACAATTATAATTAATGC
*IFNB1* (Fwd)	ACATCCCTGAGGAGATTAAGCA
*IFNB1* (Rev)	GCCAGGAGGTTCTCAACAATAG
*IFIT1* (Fwd)	CCTGAAAGGCCAGAATGAGG
*IFIT1* (Rev)	TCCACCTTGTCCAGGTAAGT
*IFIT2* (Fwd)	CTGAAGAGTGCAGCTGCCTG
*IFIT2* (Rev)	CACTTTAACCGTGTCCACCC
*IFIT3* (Fwd)	ACACAGAGGGCAGTCATGAGTG
*IFIT3* (Rev)	TGAATAAGTTCCAGGTGAAATGGC
*CXCL10* (Fwd)	GTGGCATTCAAGGAGTACCTC
*CXCL10* (Rev)	GCCTTCGATTCTGGATTCAGACA
*GAPDH* (Fwd)	ACCCAGAAGACTGTGGATGG
*GAPDH* (Rev)	TTCTAGACGGCAGGTCAGGT
pUC13-Ecogpt-EGFP-vB12-TAP	TTT*CTGCAG*AACATGGATACAGATGT (PstI)
	**TAGTCCTCGCCGCTGGCTCCCTTTTCGAACTGGGGGTGGCTCCAGCTTCCGCCTCCGCTGCCTCCTCCCTTTTCAAACTGAGGATGAGACCA**CATGCCTGCTATGTAATAACG
	**GGAGGCAGCGGAGGCGGAAGCTGGAGCCACCCCCAGTTCGAAAAGGGAGCCAGCGGCGAGGACTACAAGGATGACGATGACAAG***GCGGCCGC*GGAATCCTTCAAGTATTGTTT (NotI)
	TTT*GAATTC*GTCAATACTGACGAGATTG (EcoRI)
*B12R* sequencing primer (Fwd)	GAATGTATCTGTCACGATGTAGGA
*B12R* sequencing primer (Rev)	TCCGTAACACTACCACACTCT
*BANF1* CDS (Fwd)	AAA*GCGGCCGC*GACAACCTCCCAAAAGC (NotI)
*BANF1* CDS (Rev)	AAA*TCTAG*ATCATCACAAGAAGGCGTCG (XbaI)

a Restriction sites used are indicated in italics, and restriction enymes are indicated in parentheses following the oligonucleotide sequence where applicable. If present, sequences encoding the epitope tags are highlighted in boldface, and Kozak sequences are underlined.

### Cloning of inducible lentivirus expression vectors.

pCW57-PURO-HAB12 (11) was described previously. pCW57-PURO-HAB12-K45A, pCW57-PURO-HAB12-D156A, pCW57-PURO-HAB12-K139A, and pCW57-PURO-HAB12-K139D were produced through overlap PCR and cloning. All PCR primers are listed in Table 1. HAB12 mutant PCR products were generated by overlap PCR mutagenesis of codon-optimized HA-B12 using the primers F-HAB12 and R-HAB12 in conjunction with overlapping primers encoding the desired amino acid substitution. PCR products were digested with NheI and ligated into the first multiple cloning site of NheI-digested pCW57-PURO-MCS1-2A-MCS2. Plasmids were verified by DNA sequencing.

### Cloning of additional plasmid vectors.

pcDNA3.1+ HA-B12 (6) has been described previously. Codon-optimized, HA-tagged K45A and D156A were generated by NheI digestion of pCW57-PURO-HAB12-K45A and pCW57-PURO-HAB12D156A plasmids (described above). Restriction digest fragments were ligated into the multiple cloning site of NheI-digested pcDNA3.1+. For the pcDNA4/TO-based vectors, *B12R* from VACV strain WR was first codon optimized for expression in human cells and synthesized by GeneArt (Thermo Fisher Scientific). The B12 codon-optimized open reading frame (ORF) contained an optimal 5′ Kozak sequence, was fused to DNA encoding an N-terminal HA epitope, and was cloned between BamHI and XbaI. A NotI site+1G was inserted between the epitope tag and the ORF and generates an (Ala)_3_ linker. The pcDNA4/TO-based expression vectors for VACV protein C16 have been described (50). The ORF encoding human BAF (*BANF1* gene, NCBI GenBank accession number NM_001143985.1) was amplified with primers *BANF1* CDS (Fwd) and *BANF1* CDS (Rev) that incorporated 5′ NotI and 3′ XbaI sites, respectively, using cDNA of polyadenylated RNA from HEK293T cells. *BANF1* ORF was then subcloned into NotI and XbaI sites of pcDNA4/TO containing an optimal 5′ Kozak sequence and the nucleotide sequence encoding an N-terminal FLAG epitope upstream of the NotI site. Nucleotide sequences of the inserts in all the plasmids were verified by Sanger DNA sequencing. The primer sequences are listed in Table 1.

### Cloning of mutant VACV B12 in plasmid vectors.

pUC19-WT B12, pUC19-mut69 B12, pUC19-K45A B12, pUC19-K139A B12, pUC19-K139D B12, pUC19-D156A B12, and pUC19-M1stop B12 were generated by traditional and overlap PCR and cloning. All PCR primers are listed in Table 1. The WT B12 PCR product contains 150 bp flanking the *B12R* gene and was amplified from a VACV WR genomic DNA preparation using F-EcoRI-B11 and R-EcoRI-B13. This PCR product was digested with EcoRI and ligated into EcoRI-digested pUC19. The plasmid was verified by DNA sequencing. Mutant B12 PCR products were generated by overlap PCR on pUC19-WT B12 using F-EcoRI-B11 and reverse primers that contain the desired mutation and forward primers containing the desired mutation and R-EcoRI-B13. PCR products were digested with EcoRI and ligated into pUC19. Plasmids were verified by DNA sequencing.

### Generation of stable cells expressing WT VRK1 and mutants.

Lentiviral expression vectors for respective genes were used to generate lentiviruses as described previously (48). For transduction, cells were seeded for 50% confluence in 35-mm dishes. The next day, cell growth medium was replaced with 1 mL of lentivirus supernatant and incubated for 16 h. Medium was then replaced and incubated for an additional 24 h. Cells were then passaged in medium containing 500 μg/mL (HAP1 and VRK1KO) or 200 μg/mL (CV1) of hygromycin to select for stable lentiviral integration. Protein expression was confirmed by immunoblotting using mouse anti-FLAG M2 (Sigma) antibody. For generation of double-transduced HA-B12-expressing cells, the above selected cells underwent a second round of identical lentivirus transduction. Cells were then passaged in medium containing both hygromycin (200 μg/mL [CV1 cells] or 500 μg/mL [HAP1 cells]) and puromycin (10 μg/mL [CV1 cells] or 0.5 μg/mL [HAP1 cells]) to select for double-stable lentiviral integration. After selection, cells were induced for 24 or 48 h with 10 μg/mL doxycycline to induce protein expression. Protein expression was confirmed by immunoblotting using mouse anti-HA (BioLegend, clone 16B12) or rabbit anti-HA (Cell Signaling) antibody.

### Generation of stable cells expressing inducible B12.

The inducible lentiviral expression vector pCW57 (Addgene catalog no. 71782, a gift from Adam Karpf) was used to generate lentivirus as described previously (11). For transduction, cells were seeded for 50% confluence next day in 35-mm dishes. The next day, cell growth medium was replaced with 1 mL of lentivirus supernatant and incubated for 16 h. Medium was then replaced and incubated for an additional 24 h. Cells were then passaged in medium containing 0.5 μg/mL (HAP1, VRK1KO, and HFF-TERT) or 10 μg/mL (CV1) of puromycin to select for stable lentiviral integration. After selection, cells were induced for 24 to 48 h, as indicated, with 10 μg/mL doxycycline to induce protein expression. Protein expression was confirmed by immunoblotting using mouse anti-HA (BioLegend, clone 16B12) or rabbit anti-HA (Cell Signaling) antibody.

### MutB12, WTeHAB12, ΔB1ΔB12, and vTAP-B12 virus generation.

The mutB12 virus was generated by replacing the mCherry gene in the *B1R* gene locus of the ΔB1mutB12 virus (6) with the VACV WR *B1R* gene using homologous recombination and standard protocols. Briefly, the *B1R* gene and 150 bp of flanking sequences from VACV WR was amplified using F-A57 and R-B2 primers. Next, CV1-3×FLAGB1 cells were infected with ΔB1mutB12 virus at MOI of 0.01, followed by transfection at 3 hpi with 1.5 μg of the above-generated, linear, PCR-purified product. Cells were harvested at 48 hpi, freeze/thawed three times, and used for titration of virus infectivity on CV1-3×FLAGB1 cells. Three rounds of recombinant virus enrichment were performed on CV1 cells seeded in 6-well dishes and infected at an MOI of 0.01. Cells were harvested at 72 hpi, frozen and thawed three times and used for virus titrations on CV1-3×FLAGB1 cells. CV1 cells in 12-well dishes were infected with 1 PFU/well and plaque picked for non-mCherry-expressing plaques (indicating successful homologous recombination). MutB12 virus was plaque purified three times and confirmed to be a pure stock by loss of mCherry expression and *B1R* gene locus DNA sequencing.

The WTeHAB12 virus was generated from VACV WR and contains an N-terminal HA tag at the endogenous locus of the *B12R* gene and was generated using homologous recombination, transient dominant selection, and standard protocols. Briefly, 300-bp flanking DNA encoding an N-terminal HA tag at the endogenous *B12R* gene locus was designed containing XhoI restriction sites and was PCR amplified using F-XhoI-B11 and R-XhoI-B12 and ligated into XhoI-digested pBSIIKS-GFP-neoR GYR-PKR (plasmid kindly provided by Jason Mercer [98]). Plasmids were verified by DNA sequencing. Next, CV1-3×FLAGB1 cells were infected with VACV WR at an MOI of 0.03, followed by transfection at 3 hpi with 3 μg of the plasmid generated as described above. At 12 hpi, the medium was changed to contain 2 mg/mL G418 (Sigma). Cells were harvested 24 h after medium change, freeze/thawed three times, and used for virus titrations on CV1-3×FLAGB1 cells. Two rounds of recombinant virus enrichment were performed on CV1-3×FLAGB1 cells seeded in 6-well dishes, and samples were infected at an MOI of 0.03 in the presence of 2 mg/mL G418 to obtain a GFP^+^ virus population. Cells were harvested at 48 hpi, freeze/thawed three times, and used for virus titrations on CV1-3×FLAGB1 cells. Next, one round of selection was performed on CV1-3×FLAGB1 cells seeded in 6-well dishes and infected at an MOI of 0.03 in the presence of 100 ng/mL coumermycin A1 (Sigma) to obtain a GFP^−^ virus population. Cells were harvested at 48 hpi, freeze/thawed three times and used for virus titrations on CV1-3×FLAGB1 cells. CV1 cells in 12-well dishes were infected with 1 PFU/well and plaque picked for non-GFP-expressing plaques. WTeHAB12 virus was plaque purified three times and confirmed to be a pure stock by loss of GFP expression and *B12R* gene locus DNA sequencing.

The ΔB1ΔB12 virus contains a stop codon in place of the initiating methionine codon of the *B12R* gene and was generated from the ΔB1 virus (48) using homologous recombination and standard protocols. Briefly, CV1-3×FLAGB1 cells were infected with ΔB1 virus at an MOI of 0.1 followed by transfection of 3 μg of pUC19-M1stop B12 (described above) linearized with HindIII at 3 hpi. Cells were harvested at 48 hpi, frozen and thawed three times, and used for virus titrations on CV1-3×FLAGB1 cells. Two rounds of recombinant virus enrichment were performed on CV1 cells seeded in 6-well dishes and infected at an MOI of 0.01. Cells were harvested at 48 hpi, frozen and thawed three times, and used for virus titrations on CV1-3×FLAGB1 cells. ΔB1ΔB12 virus was plaque-purified three times and confirmed to be a pure stock by *B12R* gene locus DNA sequencing.

vTAP-B12 was generated through restoration in vΔB12 (8) of B12 expression fused to an N-terminal TAP tag under the control of its natural promoter, by means of the transient dominant selection method (99). The *B12R* ORF fused to the sequence coding an N-terminal TAP tag was also amplified by overlapping PCR, including about 250 bp upstream and downstream of the ORF, and inserted into the PstI and BamHI sites of pUC13-Ecogpt-eGFP plasmid, containing the Escherichia coli guanylphosphoribosyltransferase (Ecogpt) gene fused in-frame with the enhanced green fluorescent protein (eGFP) gene under the control of the VACV 7.5K promoter (99). The TAP tag consisted of two copies of the Strep-tag II epitope and one copy of the FLAG epitope. The derived plasmid was transfected into CV1 cells that had been infected with vΔB12 at 0.1 PFU/cell for 1 h. After 48 h, progeny viruses that incorporated the plasmid by recombination and expressed the Ecogpt-eGFP were selected and plaque purified three times on monolayers of BS-C-1 cells in the presence of mycophenolic acid (25 μg/mL), supplemented with hypoxanthine (15 μg/mL) and xanthine (250 μg/mL). The intermediate recombinant virus was submitted to three additional rounds of plaque purification in the absence of the selecting drugs, and GFP-negative plaques were selected. Under these conditions, progeny viruses can undergo a second recombination that results in loss of the Ecogpt-eGFP cassette concomitantly with either incorporation of the desired mutation (called vTAP-B12) or reversal to parental genotype (vΔB12). Viruses were analyzed by PCR to identify recombinants containing either TAP-B12 or ΔB12 alleles, and lacking the Ecogpt-eGFP cassette.

### B12 marker rescue assay.

CV1-3×FLAGB1 cells were seeded in 6-well plates and infected with ΔB1 virus at an MOI of 0.1 followed by transfection at 3 hpi with 3 μg of HindIII-linearized pUC19-WT B12, pUC19-mut69 B12, pUC19-K45A B12, pUC19-D156A B12, pUC19-K139A B12, pUC19-K139D B12, or pUC19-M1stop B12. Cells were harvested at 48 hpi, frozen and thawed three times, and used for virus titrations on CV1-3×FLAGB1 cells. Two rounds of virus enrichment were performed on CV1 cells seeded in 6-well dishes and infected at an MOI of 0.01. Cells were harvested at 48 hpi, frozen and thawed three times, and used for virus titrations on CV1-3×FLAGB1 and CV1 cells.

### DNA purification and qPCR.

VACV DNA was extracted using a GeneJET whole blood genomic DNA purification minikit (Thermo Scientific) according to the manufacturer’s protocol. VACV DNA qPCR was performed using iTaq Universal SYBR green Supermix (Bio-Rad) as described previously (51). Serial dilutions were included in each qPCR run to develop a standard curve and determine the PCR efficiency of the primer sets in each experiment. qPCR analysis was performed using 10 ng of DNA and 1 μM concentrations of each primer. Viral DNA quantification used primers F-HA and R-HA. The primers are listed in Table 1.

### Immunoblotting.

To evaluate protein expression, cells were harvested, pelleted, and suspended in sodium dodecyl sulfate (SDS) sample buffer supplemented with protease (Pierce Mini; Fisher) and phosphatase (Pierce; Fisher) inhibitors, trypsin serine protease inhibitor (phenylmethylsulfonyl fluoride; Fisher), and nuclease (Pierce universal nuclease). Sample lysates were boiled for 5 min at 95°C and resolved by SDS-PAGE (12 or 18% acrylamide) and transferred to polyvinylidene difluoride (Bio-Rad). The antibodies and the respective concentrations used are shown in Table 2. Membranes were blocked in 5% milk/1× TBST, incubated with antibodies diluted in 1% milk/1× TBST, and finally washed with 1× TBST. Membranes were developed with SuperSignal West PICO ECL (Fisher) or SuperSignal West FEMTO ECL (Fisher). Quantification of chemiluminescence signals were performed using Bio-Rad ImageLab software, and images were obtained using film and developer.

**TABLE 2:** Antibody sources and dilutions

Antibody	Source	Clone	Dilution	Catalog no.
Primary antibodies				
Rabbit polyclonal anti-BAF	Atlas Antibodies	BANF1	1:1,000	HPA039242
Mouse monoclonal anti-BAF	Santa Cruz Biotechnology	A-11	1:500	sc-166324
Rabbit polyclonal anti-BAF rabbit (total)	Laboratory of Matthew Wiebe	NA^*a*^	1:3,000	NA
Rabbit polyclonal anti-BAF rabbit (phosphorylated)	Laboratory of Matthew Wiebe	NA	1:1,000	NA
Rabbit polyclonal anti-D8	Laboratory of Geoffrey Smith	NA	1:1,000	NA
Rabbit polyclonal anti-F17	Laboratory of Paula Traktman	NA	1:5,000	NA
Mouse monoclonal anti-FLAG	Sigma Aldrich	M2	1:5,000	F3165
Rabbit polyclonal anti-FLAG	Sigma Aldrich		1:5,000	F7425
Rabbit polyclonal anti-GAPDH	Santa Cruz Biotechnology	FL-335	1:500	sc-25778
Mouse monoclonal anti-GAPDH	Sigma Aldrich	71.1	1:1,000	G8795
Rabbit polyclonal anti-H2B	Santa Cruz Biotechnology	FL-126	1:500	sc-10818
Rabbit polyclonal anti-H3	Cell Signaling	D1H2	1:1,000	4499S
Rabbit polyclonal anti-H3	Cell Signaling		1:1,000	9715
Rabbit polyclonal anti-H3Ser10(P)	Cell Signaling		1:400	3377S
Mouse monoclonal anti-HA	BioLegend	16B12	1:1,000	901501
Rabbit polyclonal anti-HA	Cell Signaling	C29F4	1:1,000	3724S
Rabbit polyclonal anti-HA	Sigma Aldrich		1:1,000	H6908
Rabbit polyclonal anti-I3	Laboratory of Paula Traktman		1:7,500	NA
Mouse monoclonal anti-IFIT2	Santa Cruz Biotechnology	F-12	1:500	sc-390724
Mouse monoclonal anti-IFIT3	Santa Cruz Biotechnology	B-7	1:500	sc-393512
Rabbit polyclonal anti-Ku70	Cell Signaling	D10A7	1:1,000	4588S
Mouse monoclonal anti-Lamin A/C	Cell Signaling	4C11	1:1,000	4777S
Mouse monoclonal anti-Lamin A/C	Abcam	131C3	1:1,000	ab8984
Rabbit polyclonal anti-p53	Cell Signaling	Z5F	1:1,000	2527S
Mouse monoclonal anti-β-tubulin I	Sigma Aldrich	SAP.4G5	1:10,000	T7816
Rat monoclonal anti-α-tubulin	Serotec	YL1/2	1:10,000	MCA77G
Mouse monoclonal anti-VRK1	Abcam	5D1	1:1,000	ab171933
				
Secondary antibodies				
Goat anti-mouse IgG HRP conjugate	BioRad		1:20,000	170-6516
Goat anti-rabbit IgG HRP conjugate	BioRad		1:20,000	170-6515
Alexa Fluor 647 goat anti-rabbit IgG	Life Technologies		1:400	A21245
Alexa Fluor 488 goat anti-mouse IgG	Life Technologies		1:400	A11029
IRDye 680RD-conjugated goat anti-rabbit IgG	LI-COR		1:10,000	926-68071
IRDye 680LT-conjugated goat anti-mouse IgG	LI-COR		1:10,000	926-68020
IRDye 800CW-conjugated goat anti-rabbit IgG	LI-COR		1:10,000	926-32211
IRDye 800CW-conjugated goat anti-mouse IgG	LI-COR		1:10,000	926-32210
IRDye 680LT-conjugated goat anti-rat IgG	LI-COR		1:10,000	926-68029

a NA, not applicable.

For estimation of B12 half-life and stability, cells were treated with cycloheximide (25 μg/mL; Sigma-Aldrich) for the indicated lengths of time, and sample lysates were harvested according to the sample collection protocol described above.

Alternatively, cells were washed with PBS and lysed on ice with cell lysis buffer (50 mM Tris-HCl [pH 8.0], 150 mM NaCl, 1 mM EDTA, 10% [vol/vol] glycerol, 1% [vol/vol] Triton X-100, and 0.05% [vol/vol] Nonidet P-40 [NP-40]), supplemented with protease (cOmplete Mini; Roche) and phosphatase (PhosSTOP; Roche) inhibitors, for 20 min. Lysed cells were scraped, and lysates were clarified to remove insoluble material by centrifugation at 17,000 × *g* for 15 min at 4°C. The protein concentration in the cell lysate was determined using a bicinchoninic acid (BCA) protein assay kit (Pierce). After mixing with 5× SDS gel loading buffer (250 mM Tris-HCl [pH 6.8], 500 mM β-mercaptoethanol, 10% [wt/vol] SDS, 50% [vol/vol] glycerol, and 0.5% [wt/vol] bromophenol blue) and boiling at 100°C for 5 min, equivalent amounts of protein samples (10 to 15 μg/well) were analyzed by SDS-PAGE and transferred onto nitrocellulose membranes (GE Healthcare). Membranes were blocked at room temperature with 5% (wt/vol) nonfat milk in Tris-buffered saline (TBS) containing 0.1% (vol/vol) Tween 20 (TBST). To detect the expression of the protein, the membranes were incubated with specific primary antibodies diluted in blocking buffer at 4°C overnight. After a washing step with TBST, the membranes were probed with fluorophore-conjugated secondary antibodies (LI-COR Biosciences) diluted in 5% (wt/vol) nonfat milk at room temperature for 1 h. After the washing, the membranes were imaged using the Odyssey CLx imaging system (LI-COR Biosciences) according to the manufacturer’s instructions. For quantitative analysis of protein levels, the band intensities on the immunoblots were quantified using Image Studio software (LI-COR Biosciences).

### Subcellular fractionation assay.

CV1-derived and HapI-derived cells were infected as indicated and then fractionated into soluble cytoplasmic (Cyto.), membrane (Memb.), nuclear (Nuc.), chromatin-bound (Chrom.) and cytoskeletal (Cytoskel.) fractions using a subcellular protein fractionation kit for cultured cells (Thermo Scientific, catalog no. 78840) according to the manufacturer’s instructions with the addition of phosphatase inhibitors. Lamin A/C was used as a nuclear protein control that is present in soluble fractions and fractions bound to the chromatin and cytoskeleton, along with H2B that localizes specifically to the chromatin nuclear fraction. GAPDH (glyceraldehyde-3-phosphate dehydrogenase) was used as a predominant cytosolic and minor membrane-associated protein control. Sample lysates were resolved by SDS-PAGE, transferred to polyvinylidene difluoride (Bio-Rad), and developed as described above.

### Soluble and insoluble cell fractionation assay.

Totals of 1.0 × 10^6^ CV1 or A549 cells or 2.57 × 10^6^ HAP1 cells, uninfected or infected with VACV WR (MOI = 5), A549 cells infected with WT or mutB12 virus with or without 50 μM AraC, or HAP1 and HAP1-VRK1KO cells stably expressing HAB12-WT, HAB12-K45A, HAB12-K139A, HAB12-K139D, or HAB12-D156A treated with 10 μg/mL doxycycline for 24 h were lysed at 7 hpi with 200 μL of lysis buffer containing 50 mM Tris-HCl (pH 7.4), 150 mM NaCl, and 1% Triton X-100 and supplemented with protease and phosphatase inhibitors and nuclease (Pierce Universal Nuclease) for 20 min at 4°C. Cell lysates were then centrifuged at 15,000 rpm at 4°C for 10 min. The supernatant was removed as the soluble cell lysate sample for Western blot analysis. The remaining pellet was used as the insoluble cell lysate sample and treated with nuclease and resuspended in 200 μL of SDS sample buffer supplemented with protease and phosphatase inhibitors. Sample lysates were resolved by SDS-PAGE, transferred to polyvinylidene difluoride (Bio-Rad), and developed as described above.

Alternatively, HEK293T and HeLa cells grown in 6-well dishes were infected with vΔB12 or vTAP-B12 (MOI = 5) for 4, 8, 12, and 24 h, or HEK293T cells were transfected overnight with plasmids expressing FLAG-tagged BAF in combination with HA-tagged GFP, C16, or B12 (1.5 μg/well of each plasmid using polyethylenimine [PEI; Polysciences], 2 μL of 1 mg/mL stock per μg of plasmid DNA). Cells were washed with PBS and lysed on ice with cell lysis buffer (50 mM Tris-HCl [pH 8.0], 150 mM NaCl, 1 mM EDTA, 10% [vol/vol] glycerol, 1% [vol/vol] Triton X-100, 0.05% [vol/vol] NP-40), supplemented with protease (complete Mini; Roche) and phosphatase (PhosSTOP; Roche) inhibitors, for 20 min. Lysed cells were scraped, and lysates were centrifuged at 17,000 × *g* for 15 min at 4°C. Supernatants containing soluble proteins were transferred to fresh tubes and mixed with 5× SDS gel loading buffer. Insoluble pellets were solubilized with SDS gel loading buffer (50 mM Tris-HCl [pH 6.8], 100 mM β-mercaptoethanol, 2% [wt/vol] SDS, 10% [vol/vol] glycerol, 0.1% [wt/vol] bromophenol blue) by rotation for 1 h at room temperature. Before mixing with SDS-gel loading buffer, the protein concentration in the soluble fractions from infected cells was determined by a BCA assay, and equivalent amounts of soluble and insoluble fraction were analyzed by SDS-PAGE and immunoblotting.

HEK293T cells were also fractionated following an adapted protocol (24, 100). Cells were washed twice with PBS, scraped in ice-cold fractionation buffer (20 mM Tris-HCl [pH 7.4], 5 mM MgCl_2_, 5 mM dithiothreitol [DTT], 150 mM KCl, 0.5% [vol/vol] Triton X-100) supplemented with protease and phosphatase inhibitors, and allowed to swell for 10 min on ice. After the addition of sucrose to a final concentration of 10% (wt/vol), lysates were spun at 1,000 × *g* for 10 min at 4°C. Supernatants containing the soluble proteins were transferred to fresh tubes, and the insoluble material was washed once in fractionation buffer, treated with 125 U of Benzonase (Millipore) for 10 to 15 min at 37°C under agitation, and spun at 1,000 × *g* for 10 min at 4°C to obtain the soluble fraction after nuclease treatment (“soluble + nuclease”). The insoluble material after nuclease treatment was washed in fractionation buffer and incubated in fractionation buffer supplemented with 0.5 M NaCl for 20 min at 4°C under agitation. Samples were spun 10,000 × *g* for 10 min at 4°C to obtain the soluble fraction after high-salt treatment (“soluble + high salt”). All fractions were mixed with 5× SDS gel loading buffer before immunoblotting.

### Salt fractionation of nucleus assay.

Salt fractionation of nuclei was adapted from established protocols (43–45). Briefly, HapI-derived cells were infected as indicated, treated with 50 μM AraC (Sigma) were harvested at 7 hpi. Cell pellets were resuspended in 250 μL of ice-cold buffer I (0.32 M sucrose, 60 mM KCl, 15 mM NaCl, 5 mM MgCl_2_, 0.1 mM EGTA, 15 mM Tris [pH 7.5], 0.5 mM DTT, 0.1 mM phenylmethylsulfonyl fluoride [PMSF], and protease inhibitors). To dissolve the plasma membrane, we added 250 μL of ice-cold buffer I supplemented with 0.1% IGEPAL and incubated the samples on ice for 10 min. One-tenth of a 500-μL volume was set aside for Western blot analysis as the cell lysate sample. The remaining 450 μL of nuclei was layered on 1 mL of ice-cold buffer II (1.2 M sucrose, 60 mM KCl, 15 mM NaCl, 5 mM MgCl_2_, 0.1 mM EGTA, 15 mM Tris [pH 7.5], 0.5 mM DTT, 0.1 mM PMSF, and protease inhibitors) and centrifuged for 20 min at 10,000 × *g* and 4°C. The pelleted nuclei were resuspended in 50 μL of buffer III (10 mM Tris [pH 7.4], 2 mM MgCl_2_, 0.1 mM PMSF) supplemented with 5 mM CaCl_2_, and the DNA was digested to mononucleosomes by the addition of 1 U of MNase (Sigma-Aldrich). The reaction mixture was incubated at 37°C for 30 min and then stopped by adding 3.3 μL of 0.1 M EGTA. The samples were centrifuged for 10 min at 350 × *g* and 4°C, and the supernatants were set aside for Western blot analysis as a nuclear digest sample. The pellet was resuspended in 50 μL of buffer IV (70 mM NaCl, 10 mM Tris [pH 7.4], 2 mM MgCl_2_, 2 mM EGTA, 0.1% Triton X-100, 0.1 mM PMSF) with 150 mM NaCl and rotated for 30 min at 4°C. The sample was centrifuged for 10 min at 350 × *g* at 4°C, and the supernatant was collected for Western blot analysis as the first salt fractionation sample. This step was repeated for salt concentrations in buffer IV of 300 and 600 mM. The final pellet was resuspended in 50 μL of SDS sample buffer. Sample lysates were subjected to immunoblot analysis as described above. Lamin A/C was used as a nuclear matrix marker, and histone H3 was used as a heterochromatin marker.

### Protein immunoprecipitations.

To study the interaction between WT B12, B12 mutants, and VRK1, 5.0 × 10^6^ of CV1-CTRL, CV1-HAB12-WT, CV1-HAB12-K45A, CV1-HAB12-D156A, CV1-HAB12-K139A, and CV1-HAB12-K139D cells treated with 10 μg/mL doxycycline for 24 h were harvested in cold PBS and pelleted. Cells were lysed with 1 mL of lysis buffer containing 50 mM Tris-HCl (pH 7.4), 150 mM NaCl, and 1% Triton X-100 and supplemented with protease and phosphatase inhibitors and nuclease (Pierce universal nuclease) for 20 min at 4°C. Cell lysates were then centrifuged at 15,000 rpm at 4°C for 10 min. The 1-mL supernatant was removed and added to 2 μL of 0.5 M EDTA. Then, 50-μL portions were set aside as input samples for Western blot analysis. The remaining 950 μL was incubated with 10 μL of anti-HA magnetic beads (Cell Signaling) overnight at 4°C. Beads were washed four times with 1 mL of 1× TBST each, and immunoprecipitated proteins were eluted with 40 μL of SDS sample buffer and boiling at 95°C for 10 min. Sample lysates were resolved on SDS-PAGE gels, transferred to polyvinylidene difluoride (Bio-Rad), and developed as described above.

Alternatively, to study the interaction between the WT B12, K45A, and D156A B12 strains and VRK1, 293T cells were seeded in 6-well plates for 80% confluence. The next day, the cells were transfected per well with 4 μL of Lipofectamine 2000 and 2 μg of pcDNA3.1+ HAB12-WT, pcDNA3.1+ HAB12-K45A, or pcDNA3.1+ HAB12-D156A. Cells were lysed at 48 h posttransfection in the lysis solution described above under bead incubation conditions. Sample lysates were subjected to immunoblot analysis.

To study the interaction between VRK1 mutants and B12, 6.9 × 10^6^ stably expressing HAP1-VRK1KO-CTRL, HAP1-VRK1KO-3×FLAGVRK1-WT, HAP1-VRK1KO-3×FLAGVRK1 1-369, HAP1-VRK1KO-3×FLAGVRK1 ΔBasic, HAP1-VRK1KO-3×FLAGVRK1 ΔBasicΔNLS, and HAP1-VRK1KO-3×FLAGVRK1 1-331 cells were infected with WR-HAB12 virus (MOI = 5). At 7 hpi, the cells were lysed with 1 mL of buffer containing 50 mM Tris-HCl (pH 7.4), 150 mM NaCl, and 1% Triton X-100 and supplemented with protease, phosphatase inhibitors, and nuclease (Pierce universal nuclease) for 20 min at 4°C. The cell lysates were then centrifuged at 15,000 rpm at 4°C for 10 min. The 1-mL supernatant was removed and added to 2 μL of 0.5 M EDTA. Then, 50-μL portions were set aside as input samples for Western blot analysis. The remaining 950 μL was incubated with 10 μL of anti-FLAG M2 magnetic beads (Thermo Fisher) overnight at 4°C. The beads were washed four times with 1 mL of 1× TBS each, and the immunoprecipitated proteins were eluted with 40 μL of SDS sample buffer with boiling at 95°C for 10 min prior to immunoblot analysis.

### Immunofluorescence assay.

Cells were plated on glass 4-well chamber slides (Fisher) for 30% confluence and treated with 10 μg/mL doxycycline for 24 h. The cells were fixed with 4% paraformaldehyde (Alfa Aesar) in 1× PBS for 15 min, washed twice with 1× TBS, permeabilized with 0.2% Triton X-100 (Sigma) in 1× TBS for 10 min, and washed twice with 1× TBS. Primary antibodies were incubated with cells for 2 h at room temperature following dilutions in 1× TBS and washed three times with 1× TBS. Secondary antibodies with conjugated fluorophore were diluted in 1× TBS, incubated with cells for 1 h at room temperature in the dark, and washed three times with 1× TBS. DAPI (4′,6′-diamidino-2-phenylindole) nuclear stain (1 mg/mL) was added to cells at a 1:1,000 dilution in 1× TBS, incubated with cells for 1 min at room temperature in the dark, and washed three times with 1× TBS. The cells were mounted with 60-mm coverslips (Fisher, 12-548-5P) and Fluoromount-G (Southern Biotech). Fluorescence images were acquired on an inverted confocal microscope (Nikon A1R) and edited with ImageJ software.

### Nucleic acid transfection for immune stimulation.

HFF-TERT-EV and HFF-TERT-HA-B12 cells in 24-well plates were induced overnight with 10 μg/mL doxycycline (Melford, UK) to induce the expression of B12. The next day, cells were transfected with HT-DNA (1 μg/mL) or high-molecular-weight poly(I·C) (0.5 μg/mL) or mock treated with TransIT-LT1 (Mirus, 2 μL per μg of nucleic acid) in a final volume of 0.6 mL. After 6 and 24 h of transfection, the cells were harvested for mRNA or protein expression analyses.

### Reverse transcription and quantitative PCR.

To analyze mRNA expression, RNA was extracted using RNeasy minikit (Qiagen), and complementary DNA (cDNA) was synthesized using SuperScript III reverse transcriptase (Invitrogen) and oligo(dT) primers (Thermo Scientific) according to the instructions of the respective manufacturers. The mRNA levels of *IFIT1*, *IFIT2*, *IFIT3*, *CXCL10*, and *GAPDH* were quantified by quantitative PCR using gene-specific primer sets, Fast SYBR green master mix (Applied Biosystems), and the ViiA 7 real-time PCR system (Life Technologies). The oligonucleotide primers used for qPCR analysis of gene expression are listed in the Table 1. The mRNA expression was calculated by the 2^–Δ^*^CT^* method using *GAPDH* as the housekeeping control gene. To analyze *IFNB1* expression, RNA samples were treated prior to cDNA synthesis with DNase I (New England Biolabs) according to the manufacturer’s instructions.

### Statistics.

All experiments were repeated at least in biological triplicate unless otherwise indicated, and graphed data represent the means of all experimental replicates. Error bars represent standard deviations from the mean viral titer, viral DNA accumulation, and protein quantification. The *P* values were calculated by using two-tailed Student *t* tests as appropriate using Prism version 8.00 for Windows (GraphPad). Representative immunoblots are shown.
